# Duplication and expression of horizontally transferred polygalacturonase genes is associated with host range expansion of mirid bugs

**DOI:** 10.1186/s12862-019-1351-1

**Published:** 2019-01-09

**Authors:** Pengjun Xu, Bin Lu, Jinyan Liu, Jiangtao Chao, Philip Donkersley, Robert Holdbrook, Yanhui Lu

**Affiliations:** 1 Tobacco Research Institute, Chinese Academy of Agricultural Sciences, Qingdao, 266101 People’s Republic of China; 2 State Key Laboratory for Biology of Plant Diseases and Insect Pests, Institute of Plant Protection, Chinese Academy of Agricultural Sciences, Beijing, 100193 People’s Republic of China; 3 Lancaster Environment Centre, Lancaster University, Lancaster, LA1 4YQ UK; 4 Department of Herpetology, Chengdu Institute of Biology, Chinese Academy of Sciences, Chengdu, Sichuan 610041 People’s Republic of China

**Keywords:** Polygalacturonase, Gene duplication, Molecular evolution, Expression, Host range expansion

## Abstract

**Backgroud:**

Horizontal gene transfer and gene duplication are two major mechanisms contributing to the evolutionary adaptation of organisms. Previously, polygalacturonase genes (PGs) were independently horizontally transferred and underwent multiple duplications in insects (e.g., mirid bugs and beetles). Here, we chose three phytozoophagous mirid bugs (*Adelphocoris suturalis*, *A. fasciaticollis*, *A. lineolatus*) and one zoophytophagous mirid bug (*Nesidiocoris tenuis*) to detect whether the duplication, molecular evolution, and expression levels of PGs were related to host range expansion in mirid bugs.

**Results:**

By RNA-seq, we reported 30, 20, 19 and 8 PGs in *A. suturalis*, *A. fasciaticollis*, *A. lineolatus* and *N. tenuis*, respectively. Interestingly, the number of PGs was significantly positive correlation to the number of host plants (*P* = 0.0339) in mirid bugs. Most PGs (> 17) were highly expressed in the three phytozoophagous mirid bugs, while only one PG was relatively highly expressed in the zoophytophagous mirid bug. Natural selection analysis clearly showed that a significant relaxation of selection pressure acted on the PGs in zoophytophagous mirid bugs (K = 0.546, *P* = 0.0158) rather than in phytozoophagous mirid bugs (K = 1, *P* = 0.92), suggesting a function constraint of PGs in phytozoophagous mirid bugs.

**Conclusion:**

Taken together with gene duplication, molecular evolution, and expression levels, our results suggest that PGs are more strictly required by phytozoophagous than by zoophytophagous mirid bugs and that the duplication of PGs is associated with the expansion of host plant ranges in mirid bugs.

**Electronic supplementary material:**

The online version of this article (10.1186/s12862-019-1351-1) contains supplementary material, which is available to authorized users.

## Background

Horizontal gene transfer (HGT) and gene duplication are two major mechanisms contributing to the evolutionary adaptation of organisms [1–6]. HGT refers to the transfer of genetic materials between species with reproductive isolation, which was first reported more than 70 years ago and has been found to help organisms rapidly adapt to novel environments [1, 7–9]. Gene duplication is one of the main sources of functional diversity at the genotypic level, contributing to the origin of new genes, the evolution of new gene function, and in certain instances to the evolution of organisms [10–12]. Over the last few decades, these two evolutionary adaptations have been well investigated, but the adaptation by duplication of horizontally transferred genes has rarely been reported.

As one of a group of plant cell wall-degrading enzymes (PCWDEs), polygalacturonase (PG) is ubiquitous in fungi, bacteria, and plants, catalyzing hydrolysis of α-1, 4-glycosidic linkages in polygalacturonic (pectic) acid [13]. Interestingly, PGs have also been detected in some insects from Hemiptera and Coleoptera, such as mirid bugs and leaf beetles [14–16]. Biochemically, PGs were first reported in the salivary glands of mirid bugs (*Lygus* spp.) [17–20]. Subsequently, the genes encoding PG proteins were cloned from mirid bugs and leaf beetles, and phylogenetic analysis suggested these genes were horizontally transferred to mirids from fungi and had undergone multiple duplications [14, 16, 21]. Plants possess cell walls made of complex composite fibers, which prevent insects from feeding on plant nutrients [22]. Thus, the duplication of digestive enzymes (PG genes) was considered as potentially expanding the host plant range of these insects [14, 15].

Previously, we demonstrated the duplication of PG genes in *Apolygus lucorum* [16]. According to host ranges, species in the Miridae (Hemiptera) were divided into two groups: one named phytozoophagy was mainly phytophagous with prey to complementand the other named zoophytophagy was predator which occasionally fed on plant resources) [23, 24]. Thus are an ideal group to test the hypothesis that adaptive evolution of organism by horizontal gene transfer and gene duplication e.g. insects expand their host plant range by PG genes which may have occurred in major phytozoophagous mirid bugs (*A. lucorum, Adelphocoris suturalis*, *A. fasciaticollis* and *A. lineolatus* [25, 26]) that ancestrally may have been soley predaceous (such as *Nesidiocoris tenuis*) [27]. Here, we determined the numbers of expressed PG genes in *A. suturalis*, *A. fasciaticollis*, *A. lineolatus* and *N. tenuis* by RNA-seq. We also investigated the expression levels of PG genes in *A. suturalis* and *N. tenuis*. Our results indicate that the phytozoophagous mirid bugs possessed more PG genes and higher expressed levels of these genes than did the one of zoophytophagous species examined, suggesting that the gene duplication of horizontally transferred PG genes may have been part of what allowed the host range expansion toward mixed phytozoophagy in mirid bugs.

## Methods

### Ethics statement

With permission, we captured the insects in experiment stations of Chinese Academy of Agricultural Sciences. No permits were required for the described insect collection and experimentation.

### Insects

Adults of *A. suturalis*, *A. fasciaticollis,* and *A. lineolatus* were collected from a cotton field at the Langfang Experimental Station of the Chinese Academy of Agricultural Sciences (Hebei Province, China) in 2015. Adults of *N. tenuis* were collected from a tobacco field at the Jimo Experimental Station of the Chinese Academy of Agricultural Sciences (Shandong Province, China) in 2014. Field-collected insects were used for genetic analyses.

### Transcriptome analysis

Compared with nymphs, adults of mirids can feed on a wider diversity of plant species because their ability to fly allows them to move from species to species as plants flower. Therefore, we chose adults to investigate the PG genes in this study. Fifty adults for each group (*A. suturalis* = 3 groups, *A. fasciaticollis* = 1 group, *A. lineolatus =* 1 group, and *N. tenuis* = 3 groups) were used to isolate total RNA with Trizol reagent (Invitrogen, Carlsbad, CA, USA) following the manufacturer’s instructions. The cDNA library was constructed, sequenced and analyzed as described by Xu et al. [28]. Briefly, the mRNA was isolated using Oligo (dT) magnetic beads, broken into short fragments and used to synthesize cDNA. The short fragments were purified with the QiaQuich PCR Purification Kit (Qiagen, Germany) and used to construct the cDNA library. The library was sequenced on an Illumina Hiseq™ platform and about 5 gigabase (Gb) of data were generated for each sample, using Majorbio (Beijing, China). Low-quality reads were deleted using Fastx-tools and clean pair-end reads were used for de novo assembly with Trinity (v2.0.6) software [29]. Contigs longer than 200 bases were used for subsequent analysis. The reads from libraries of each species were mapped to the assembled contigs using Bowtie 0.12.7 [30]. The read counts were further normalized as fragments per kilobase of exon model per million mapped reads (FPKM) values [31]. Gene expression profile was estimated using FPKM values by RSEM (v1.1.17) software with default parameters [32]. Unigenes were annotated with *blastx* BLAST based on the databases of Nr (NCBI non-redundant protein sequences) (https://www.ncbi.nlm.nih.gov/genbank/ and https://www.ncbi.nlm.nih.gov/protein/), String (Search Tool for the Retrieval of interacting Genes/Proteins) (https://string-db.org/), Swissprot (A manually annotated and reviewed protein sequence database) (http://www.ebi.ac.uk/uniprot/) and KEGG (Kyoto Encycloedia of Genes and Genomes) (https://www.genome.jp/kegg/) for functional annotation. The e-value cut-off was set at 1e-5 for further analysis.

### Identification of PG genes

To annotate the PGs in the four mirid bugs, 202 coding sequences of PGs were used as query sequences, including 188 coding sequences from Broad Institute (BI), Joint Genome Institute (JGI) and GenBank at National Center for Biotechnology Information (NCBI) [14] and 14 coding sequence from the salivary glands of *A. lucorum* from our previous work [16]. Then, BLAST was performed for searching PGs. To determine the genomic structure of PGs, we designed primers according to the reference sequences from RNA-seq to amplify PG genes in *N. tenuis* and *A. suturalis* using DNA as template (Additional file 1: Table S1). The PCR program was as follows: 30 s at 94 °C, 30 s at 55 °C, and 60 s at 72 °C, for 40 cycles.

### Sequence alignment, and phylogenetic/evolutionary analyses

Beside our self-sequenced PG genes, we also downloaded PG genes of *A. lucorum* and *Lygus lineolaris* from Genbank and then constructed a dataset containing 147 PG genes for subsequent evolutionary analysis. The sequence alignment was performed using the codon model as implemented in PRANK [33]. Phylogenetic analysis was performed using maximum likelihood (ML) method under the GTR + G substitution model [34] with 1000 replicates implemented in RAxML 7.3.2 [35].

### Test of selection

After duplication, genes tended to have a different selective pressure measured as a ratio between synonymous and non-synonymous substitution (*dn/ds*). To investigate the drive force behind the shift toward plant feeding, we searched for potential selections acting on PG genes in mirids. Here we mainly focus on the transformation between zoophytophagous (*N. tenuis*) and phytozoophagous (the other mirids). We use an ML approach [36] to test differences in selection pressure between the two feeding habits using the CODEML program implemented in the PAML 4.5 package [37]. We tested whether specific branch models were used to detect positive selection acting on the particular lineages. Four hypotheses were evaluated: (1) one *d*_*n*_*/d*_*s*_ ratio for all branches (one-ratio model; assuming that all branches have been evolving at the same rate); (2) *d*_*n*_*/d*_*s*_ ratio = 1 for all branches (neutral model; neutral evolution for all branches); (3) zoophytophagous and phytozoophagous lineages have a different *d*_*n*_*/d*_*s*_ ratio (ω_2_ and ω_1_; two ratio model; allowing a foreground branch to evolve under a different rate); and finally (4) neutral evolution for zoophytophagous lineage (ω_2_ = 1). A likelihood ratio test (LRT) was employed to determine if the alternative model, indicating positive selection, was superior to the null model. The newly developed RELAX method [38], as implemented in the program HYPHY [39], was employed to detect if the relaxation of selective strength occurred at the phytozoophagous and/or zoophytophagous lineage of PG genes and led to loss of function.

### Statistics

Statistical analyses were conducted using Relative valuation and one way ANOVA with SPSS Version 11.0 software package.

## Results

### Identification of PGs in four species of mirid bugs by transcriptome analysis

After removing adaptor sequences and low-quality reads, we obtained a total of 23.29, 4.51, 5.12, and 26.18 Gb of clean data in *A. suturalis*, *A. fasciaticollis*, *A. lineolatus,* and *N. tenuis,* respectively. An overview of the sequencing and assembly data are shown (Additional file 1: Table S2 and Additional file 2: Figures S1, S2, S3, S4). The RNA-seq data were submitted to the NCBI Gene Expression Omnibus (GEO) database (accession number: GSE90671) [40], and Sequence Read Archive (SRA) database (accession number: SRR6322944, SRR6322963, SRR6322964, SRR6322965, SRR6322463, SRR8259282, SRR8259810, SRR8259912). Using functional annotation, 29,890, 17,879, 25,604, and 39,937 genes (22.2, 30.6, 24.5 and 25.4% of transcripts) were able to be get BLAST hits using the E-value cutoff and NR database.

**Fig. 1:**
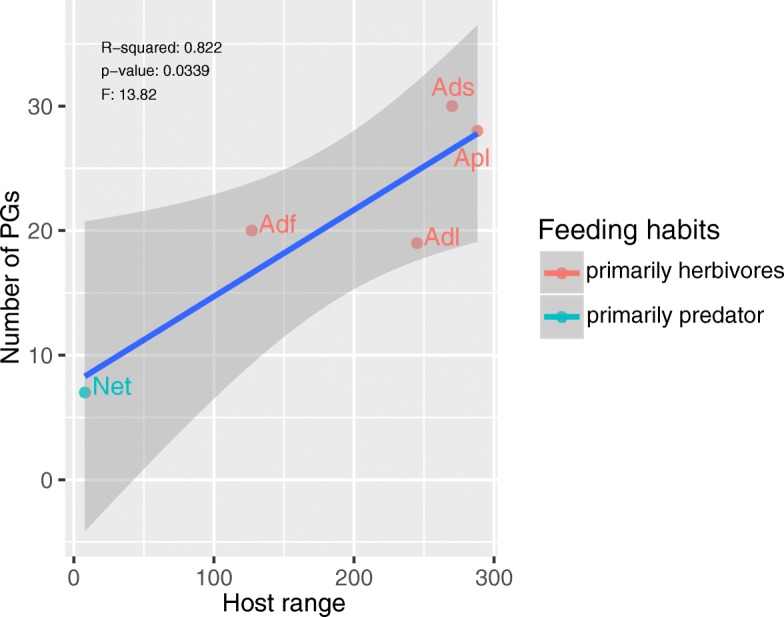
Correlation between number of polygalacturonase genes (PGs) and host plants. Apl = *Apolygus lucorum,* Ads = *Adelphocoris suturalis*, Adf = *A. fasciaticollis*, Adl = *A. lineolatus*, Net = *Nesidiocoris tenuis*

With functional annotation and a BLAST search using 202 PGs as reference sequences, we found 30, 20, 19, and 7 PGs in *A. suturalis*, *A. fasciaticollis*, *A. lineolatus,* and *N. tenuis,* respectively (Additional file 1: Table S3-S6). The known host plants of *A. suturalis*, *A. fasciaticollis*, *A. lineolatus*, *A. lucorum,* and *N. tenuis* include 270, 127, 254, 288 and 8 species, respectively [41–43]. It should be noted that without genomes for these species plus transcriptomes from different developmental stages, a thorough phylogenetic analysis will be difficult, because what is missing from the adult transcriptome cannot readily be seen. Previously, 28 PGs were reported in *A. lucorum* at NCBI, which included 14 PGs we found from the cDNA library [16]. With these data, we analyzed the correlation between the number of host plants and the number of PGs in the five mirid bugs and found a significantly positive correlation (r^2^ = 0.822, F = 13.82, *P* = 0.0339) (Fig. 1).

### Sequence alignment and phylogenetic analysis

**Fig. 2:**
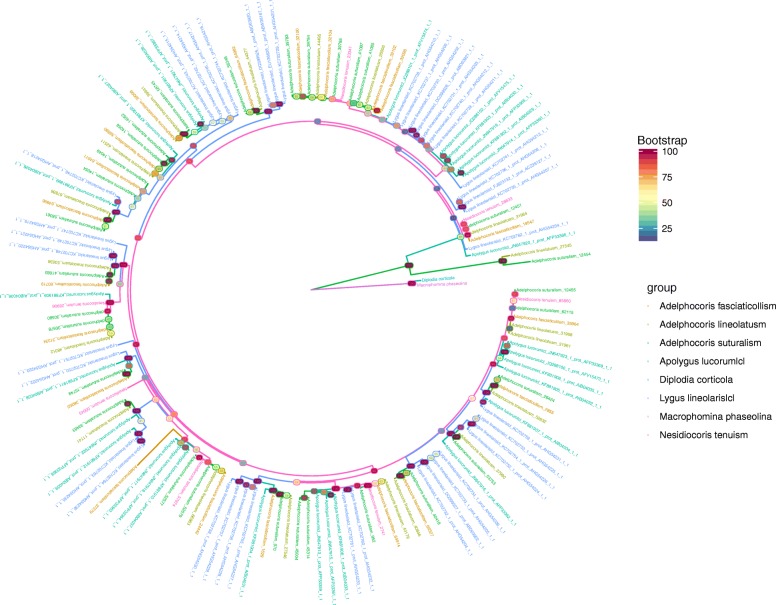
Phylogenetic tree of PGs in mirid bugs. Different species are presented in different colors. Numbers on the node are RAxML bootstrap values (Blue: low support value; Red: high support value). The GenBank accession numbers were shown or the sequences were attached (see electronic Additional file 3)

Our phylogenetic tree strongly supported all the PGs in mirid bugs being clustered together (Fig. 2; Bootstrap value = 100; Additional file 3), suggesting that the PGs of mirid bugs were derived from fungi and subsequently underwent multiple duplications after horizontally transferring into the genome of mirid bugs. Previously, no more than 50 PGs have been reported. We classified PGs of *A. lucorum* to six groups according to identities [15]; however, it was difficult to classify them clearly, using more than 100 members from seven species. To determine the gene duplication mode of PGs, we detected the genome structure of seven PGs with complete coding sequence (CDS) by designing primers and amplifying fragments using DNA as templates (six PGs in *A. suturalis* and one PG in *N. tenuis*), and only one PG in *A. suturalis* and the PG in *N. tenuis* contained one intron in the open reading frame region (Additional file 2: Figure S5).

### Natural selection analysis

The codeml branch model significantly rejected the neutral evolutionary hypothesis for the whole phylogeny of mirid PGs (Table 1, LRT = 9746.22, *p* < 0.001). Also our analysis suggests that the zoophytophagous lineages, which experienced serious loss of PGs, also evolved according to the non-neutral pattern (LRT = 41.12, *P* < 0.001). Given this result, we further specifically tested whether the selection pressure differed between predatory and phytozoophagous mirid bugs. When we set zoophytophagous lineages as foreground and phytozoophagous branches as reference, RELAX estimated the selection intensity parameter value of K = 0.546 (K = 1 is RELAX’s null hypothesis; K < 1, selection pressure been relaxed; K > 1, selection pressure been intensified), and the alternative relaxation model significantly outperformed the null model with a *P*-value = 1.58 × 10^− 4^, which is consistent with the results assessed with the likelihood ratio test. However, this was not the case when we set phytozoophagous lineages as the foreground (K = 1.0 and P-value = 0.92), which suggest that a relaxation of selection pressures occurred on the zoophytophagous branches.

**Table 1:** Selective patterns for PG genes

Model	np^a^	Ln L^b^	Estimates of ω	Models compared	LRT^c^	*P* Values
Branch model						
A: one ratio	293	−59,339.55	ω = 0.10894			
B: one ratio ω = 1	292	−64,212.66	ω = 1	B vs. A	9746.22	0.0
C: predacity branches have ω_1_, the other branches have ω_0_	294	−59,339.50	ω_1_ = 0.09379 ω_0_ = 0.10900	A vs. C	0.1	0.75
D: predacity branches have ω_1_ = 1	293	−59,360.06	ω_1_ = 1 ω_0_ = 0.10798	D vs. C	41.12	0.0

^a^Number of parameters

^b^The natural logarithm of the likelihood value

^c^Twice the log likelihood difference between the two models

### Expression analysis

**Fig. 3:**
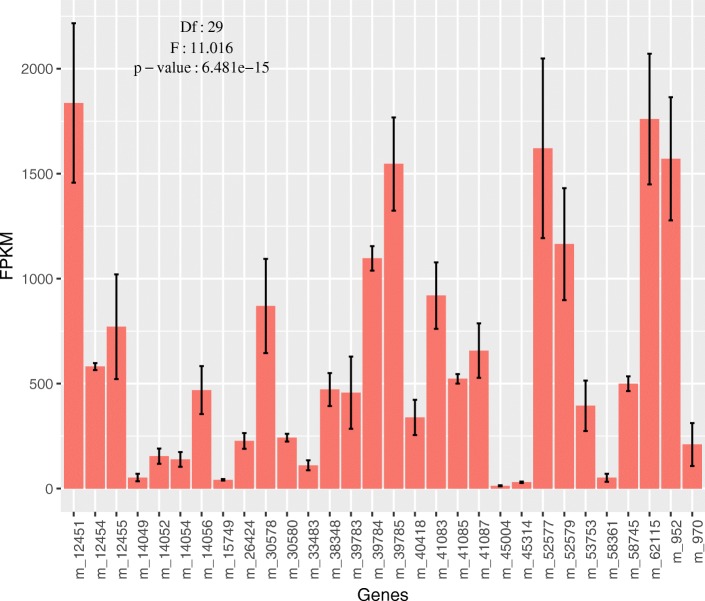
The expression levels of PGs in *Adelphocoris suturalis*. The average of FPKM values were more than 5 in the 30 PG genes suggesting a relatively high expression levels of these genes. The FPKM values were used for statistical analysis and the significance was shown. Mean ± SD

**Fig. 4:**
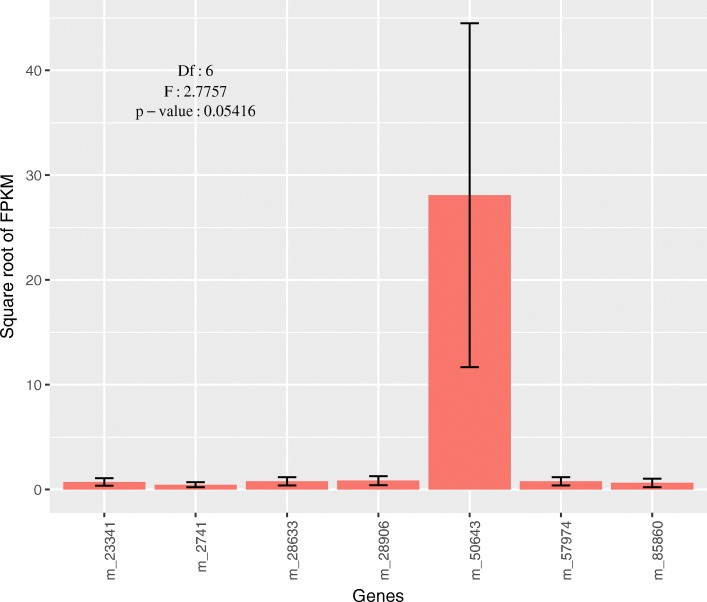
The expression levels of PGs in *Nesidiocoris tenuis*. The average of FPKM value of only one in 7 PG genes was more than 5 suggesting only one PG gene was relatively highly expressed in *N. tennuis*. The square roots of FPKM values were used for statistical analysis. Mean ± SD

To detect the mRNA level of PGs in phytozoophagous and zoophytophagous mirid bugs, we chose *A. suturalis* and *N. tenuis* to perform RNA-seq for three replicates (groups) and used the CDS sequence of PGs for counting FPKM value. In *A. suturalis*, 29 of 30 PGs were highly expressed (average FPKM value > 30) and the expression level of these 30 PGs showed statistically significant differences (d.f. = 29,60, F = 11.016, *P* = 6.481e-15) (Fig. 3). Only one in seven PGs of *N. tenuis* was highly expressed, but the expression levels were not statistically significant because of big differences among samples (d.f. = 6,14, F = 2.7757, *P* = 0.05416) (Fig. 4). In *A. fasciaticollis* and *A. lineolatus*, we sequenced one sample per species. As with PGs from the phytozoophagous *A. suturalis*, 17 of 20 PGs *A. fasciaticollis* and 16 of 17 PGs in *A. lineolatus* were highly expressed (FPKM value > 30) (Additional file 2: Figures S6, S7).

## Discussion

As barriers to plant nutrients, the diversity of cell walls may promote the adaptive evolution of pests, including insects and pathogenic microbes. Mirid bugs are responsible for serious yield loss in several economically important crops (including cotton and grapes) by causing plant stunting and fruit abscission [44]. To feed on plants, mirid bugs use horizontally transferred PG genes derived from fungi and then multiplied these genes [14, 16]. However, it remains unclear whether the gene duplication of PGs is related to the host range expansion of mirid bugs or not. The PG genes and host-range diversity of species within the family Miridae forms an ideal model to investigate the adaptive evolution of host-shift by horizontally transferred gene duplication [14, 25, 41–43]. Next generation sequencing (NGS) facilitates the investigation of these expressed PG numbers and levels [45].

### Identification of PGs in mirid bugs

In this study, we determined the expressed PG genes in three phytozoophagous and one zoophytophagous mirid bugs by RNA-seq technology. Phytozoophagous mirid bugs usually have a broad host-plant range, while zoophytophagous mirid bugs feed on only a few plants [41–43]. Interestingly, we found a significant, positive correlation between the numbers of host plants and the numbers of expressed PGs in mirid bugs, suggesting the possibility that PG gene duplication may promote host-plant expansion in mirid bugs. There are two sources for gene duplication (DNA and RNA): the first can be explained by three established models: unequal crossing-over, duplicative (DNA) transposition and polyploidization; the second is called retrotransposition, which is from mature RNA and without introns [46, 47]. To determine the duplicated model of PGs, we detected the genomic structure of seven PGs in *A. suturalis* and *N. tenuis*. One intron was found in the CDS region of PG in *N. tenuis*. There were no introns in the CDS regions of six PGs. Although we found introns in PGs of mirid bugs, we could not exclude the possibility that the duplication of PGs in mirid bugs derived from retrotransposition.

### The molecular evolution of PGs in mirid bugs

Theoretically, neutral evolution and natural selection drive ecological population divergence and the speciation of organisms [48–50]. As major digestive enzymes of plants, PGs in phytozoophagous mirid bugs might be under more selection pressure than PGs in zoophytophagous bugs. As expected, a test of selection suggested that PGs of mirid bugs were under natural selection but not neutral evolution. Although no positive selection was detected on PGs of phytozoophagous mirid bugs, our results clearly showed significantly relaxed selection in PGs of predators, suggesting a possible loss of function of PGs in zoophytophagous mirid bugs. Molecular phylogeny analysis indicated that zoophytophagous mirid bugs (e.g., *N. tenuis* from the Bryocorinae) were more ancient than phytozoophagous mirid bugs (e.g., *A. fasciaticollis*, *A. lineolatus*, *A. suturalis,* and *A. lucorum* from the Mirinae), suggesting a host-expansion from zoophytophagous to phytozoophagous mirid bugs [24]. Indeed, phytozoophagous mirid bugs also prey on insects [51]. Taken together, horizontal transfer of PGs from fungi may promote host expansion in mirid bugs: initially, mirid bugs mainly preyed on arthropods and were unable to feed on plants independently but only on plant tissues digested by fungi; subsequently, they obtained PGs from fungi by HGT and gained the capacity to feed on plants independently; finally, phytozoophagous mirid bugs might expand their host-plant ranges by multiplying the number of their PGs, while predatory mirid bugs kept low copy numbers of PGs, which underwent relaxed selection because of the largely non-phytophagous nature of the diet of these species.

### Expression patterns of PGs in mirid bugs

Adaptive evolution at a molecular level includes two mechanisms to regulate gene function: (1) nucleotide/amino acid sequence variation which could be detected with selection pressure as described above, and (2) expression levels which are essential for gene function (e.g., opsin evolution in the visual system) [52–55]. To determine whether all PGs are highly expressed and whether the expression levels of PGs were different between phytozoophagous and zoophytophagous mirid bugs, we analyzed the expression levels of PGs in *A. suturalis* (three replicates), *N. tenuis* (three replicates), *A. fasciaticollis* (no replicates) and *A. lineolatus* (no replicates) using FPKM values from of RNA-seq. Our results clearly showed that almost all PGs were highly expressed in the three phytozoophagous mirid bugs (> 17), but only one PG was relatively highly expressed in zoophytophagous mirid bugs with huge fluctuation, which most likely resulted from that PGs were needed more by phytozoophagous than zoophytophagous mirid bugs. The other reason that feeding on cotton caused the upregulation of PGs could not be completely excluded because the samples used in this study of phytozoophagous mirid bugs were collected in cotton field and the zoophytophagous mirid bugs were collected in tobacco field. Controversially, *N. tenuis* has been used as a natural enemy for pest management because of its zoophytophagy [56–58]. Taking the evidence of molecular evolution together with the mRNA expression levels of PGs, our data support the use of *N. tenuis* as a natural enemy due to the relaxed selection and only one PG expressed in *N. tenuis*, but with controlled density because of occasionally high expression level.

## Conclusions

We identified the number of expressed PGs in three phytozoophagous and one zoophytophagous mirid bug and found a significant, positive correlation between the numbers of PGs and host plants. Natural selection analysis suggested the PGs of zoophytophagous mirid bug were under a significantly relaxed selection. More than 17 PGs were highly expressed in each of the three species of phytozoophagous mirid bugs, but only one PG was relatively highly expressed in predatory mirid bugs. Taken together with evidence of gene copy number, molecular evolution and gene expression levels, our results suggested that PGs were more needed by phytozoophagous than zoophytophagous mirid bugs and the duplication of PGs promoted the host-expansion of mirid bugs. This research suggests that PGs are target genes for the management of phytozoophagous mirid bugs (e.g. RNAi).

## Additional files

Additional file 1: **Table S1.** Primers used in this study. **Table S2.** Summary of the sequence assembly after Illumina sequencing. As = *Adelphocoris suturalis*, Af = *A. fasciaticollis*, Al = *A. lineolatus*, Nt = *Nesidiocoris tenuis*. **Table S3.** Identification of polygalacturonase genes in *Adelphocoris suturalis*. **Table S4.** Identification of polygalacturonase genes in *Adelphocoris fasciaticollis*. **Table S5.** Identification of polygalacturonase genes in *Adelphocoris lineolatus*. **Table S6.** Identification of polygalacturonase genes in *Nesidiocoris tenuis*. (XLSX 24 kb)

Additional file 2: **Figure S1.** The discription of RNA-seq in *Adelphocoris suturalis*. (a) The distribution of sequences length. (b) The E-value distribution of the top matches in the nr database. (c) The species distribution of the matches in the nr database. (d) The sequence similarity distribution. **Figure S2.** The discription of RNA-seq in *Adelphocoris fasciaticollis*. (a) The distribution of sequences length. (b) The E-value distribution of the top matches in the nr database. (c) The species distribution of the matches in the nr database. (d) The sequence similarity distribution. **Figure S3.** The discription of RNA-seq in *Adelphocoris lineolatus*. (a) The distribution of sequences length. (b) The E-value distribution of the top matches in the nr database. (c) The species distribution of the matches in the nr database. (d) The sequence similarity distribution. **Figure S4.** The discription of RNA-seq in *Nesidiocoris tenuis*. (a) The distribution of sequences length. (b) The E-value distribution of the top matches in the nr database. (c) The species distribution of the matches in the nr database. (d) The sequence similarity distribution. **Figure S5.** The genome structure of polygalacturonase gene (m_50643) in *Nesidiocoris tenuis*. The intron was showed using shade. **Figure S6.** The expression levels of PGs in *Adelphocoris fasciaticollis*. **Figure S7**. The expression levels of PGs in *Nesidiocoris tenuis*. (DOCX 825 kb)

Additional file 3: The coding sequence of PGs identified in this study. (ZIP 71 kb)

## Abbreviations

BI: Broad Institute
CDS: Coding sequence
FPKM: Fragments per kilobase of exon model per million mapped reads
Gb: Gigabases
HGT: Horizontal gene transfer
JGI: Joint Genome Institute
LRT: Likelihood ratio test
ML: Maximum likelihood
NCBI: National Center for Biotechnology Information
PCWDE: Plant cell wall-degrading enzyme
PG: Polygalacturonase
