# Supplementary material for: Duplication and expression of horizontally transferred polygalacturonase genes is associated with host range expansion of mirid bugs
Source: BMC Evol Biol. 2019 Jan 9;19:12. doi: 10.1186/s12862-019-1351-1 (PMC6327464; doi:10.1186/s12862-019-1351-1)
Supplement: Supplementary file 2 — Figure S1. The discription of RNA-seq in Adelphocoris suturalis. (a) The distribution of sequences length. (b) The E-value distribution of the top matches in the nr database. (c) The species distribution of the matches in the nr database. (d) The sequence similarity distribution. Figure S2. The discription of RNA-seq in Adelphocoris fasciaticollis. (a) The distribution of sequences length. (b) The E-value distribution of the top matches in the nr database. (c) The species distribution of the matches in the nr database. (d) The sequence similarity distribution. Figure S3. The discription of RNA-seq in Adelphocoris lineolatus. (a) The distribution of sequences length. (b) The E-value distribution of the top matches in the nr database. (c) The species distribution of the matches in the nr database. (d) The sequence similarity distribution. Figure S4. The discription of RNA-seq in Nesidiocoris tenuis. (a) The distribution of sequences length. (b) The E-value distribution of the top matches in the nr database. (c) The species distribution of the matches in the nr database. (d) The sequence similarity distribution. Figure S5. The genome structure of polygalacturonase gene (m_50643) in Nesidiocoris tenuis. The intron was showed using shade. Figure S6. The expression levels of PGs in Adelphocoris fasciaticollis. Figure S7. The expression levels of PGs in Nesidiocoris tenuis. (DOCX 825 kb) [file 12862_2019_1351_MOESM2_ESM.docx]

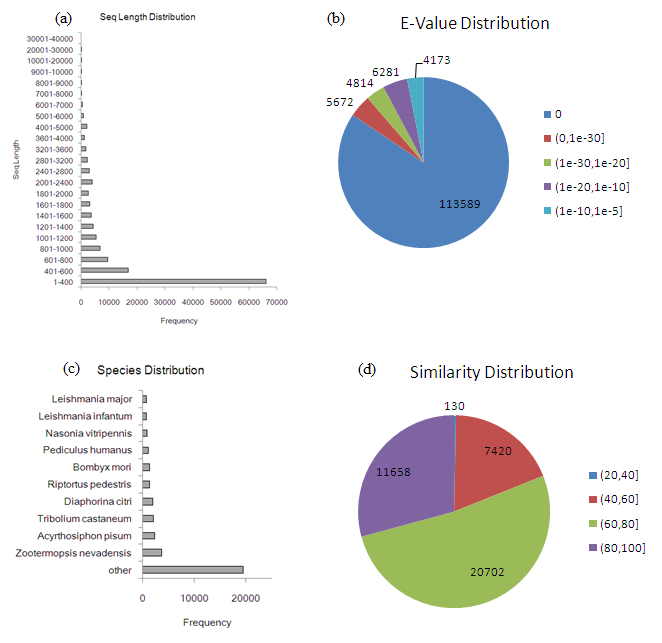


**Figure S1.** The discription of RNA-seq in *Adelphocoris suturalis*. (a) The distribution of sequences length. (b) The E-value distribution of the top matches in the nr database. (c) The species distribution of the matches in the nr database. (d) The sequence similarity distribution.


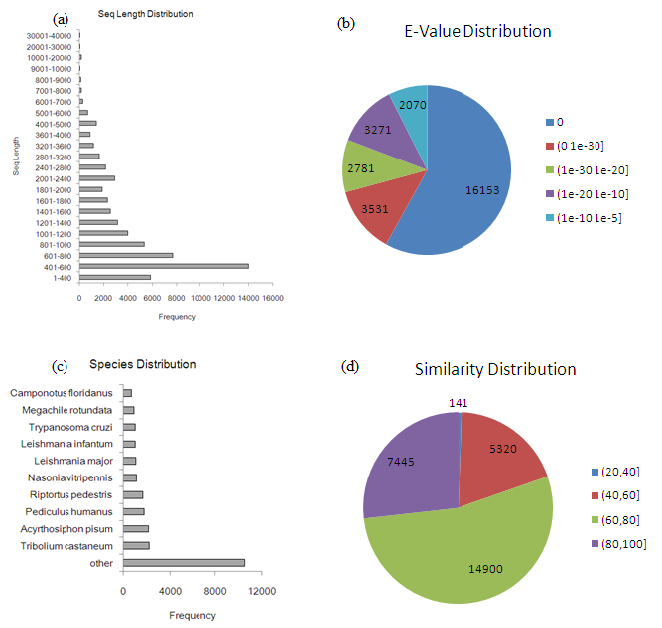


**Figure S2.** The discription of RNA-seq in *Adelphocoris fasciaticollis*. (a) The distribution of sequences length. (b) The E-value distribution of the top matches in the nr database. (c) The species distribution of the matches in the nr database. (d) The sequence similarity distribution.


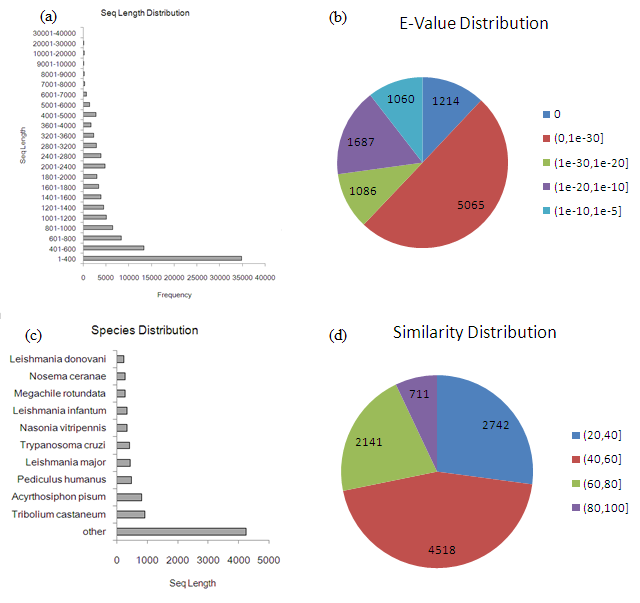


**Figure S3.** The discription of RNA-seq in *Adelphocoris lineolatus*. (a) The distribution of sequences length. (b) The E-value distribution of the top matches in the nr database. (c) The species distribution of the matches in the nr database. (d) The sequence similarity distribution.

*lineolatus*


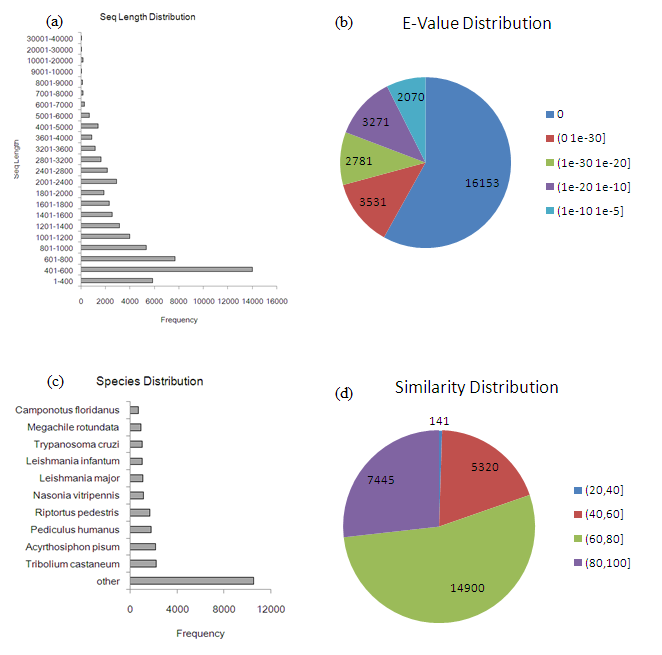


**Figure S4.** The discription of RNA-seq in *Nesidiocoris tenuis*. (a) The distribution of sequences length. (b) The E-value distribution of the top matches in the nr database. (c) The species distribution of the matches in the nr database. (d) The sequence similarity distribution.


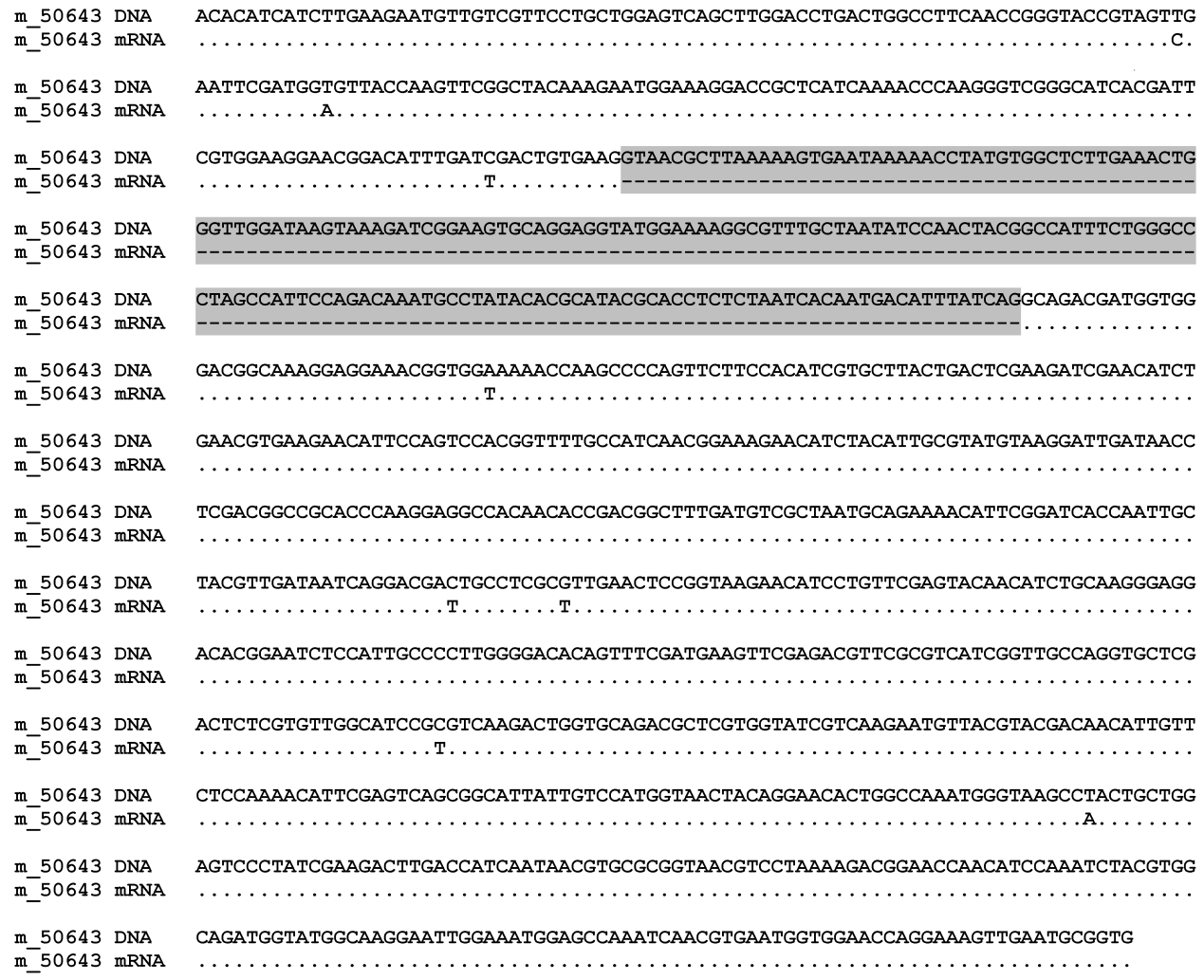


**Figure S5.** The genome structure of polygalacturonase gene (m_50643) in *Nesidiocoris tenuis*. The intron was showed using shade.


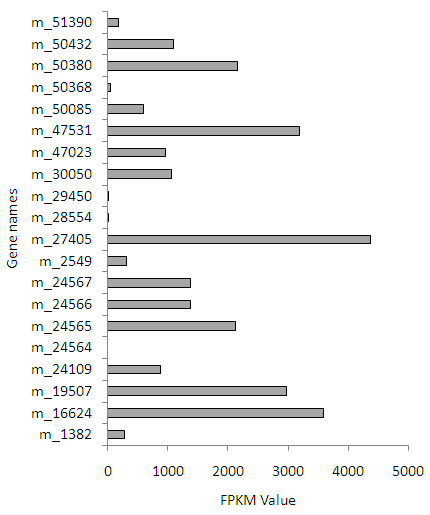


**Figure S6.** The expression levels of PGs in *Adelphocoris fasciaticollis*.


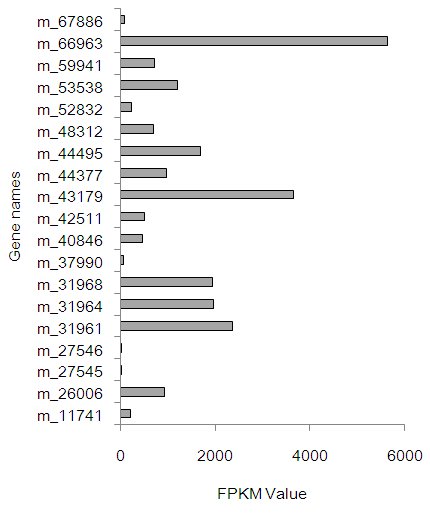


Figure S7. The expression levels of PGs in *Nesidiocoris tenuis*.
