# Supplementary material for: Duplication and expression of horizontally transferred polygalacturonase genes is associated with host range expansion of mirid bugs
Source: BMC Evol Biol. 2019 Jan 9;19:12. doi: 10.1186/s12862-019-1351-1 (PMC6327464; doi:10.1186/s12862-019-1351-1)
Supplement: Supplementary file 3 — The coding sequence of PGs identified in this study. (ZIP 71 kb) [file 12862_2019_1351_MOESM3_ESM.zip › Additional file 3-The coding sequence of polygalacturonase genes in Adelphocoris fasciaticollis.docx]

>m_1382

ATGAGGTCGGCAATTTTAGTCCTTGGAGGGGTGTTTTTCGTCCTGCACGCTGCAGTTGGT

TTCGATGTGTGGAACATGCAGCAACTGGAGGAGGCCAAGAAGACCGGCCAGAAGCTCGTT

CGAGTGAGAGATCTTCAGGTCCCTGCCGGCAAGACATTGGATTTCCAGGGCCTTGGGAAC

GGGACGACTATTGAGTTTGTTGGACGAGTTTCGTTCGGCTACAAAGAATGGAGGGGCCCT

CTGATCATCATCAAAGGCGCCAACTACGTCGTCAAAGGTCTTCCAGGACACGTTATCGAC

GGCGAAGGCCAGCGCTGGTGGGACGGGCTTGGTGGTATCACTGGAGGCAAAATCAAGCCA

GCTCCCTTCATCTACATGCAGCTTGAGAACTCCTACGTCAACGACTTGGTATTCAAGAAT

GCACCTATGACCGTCATGGCTATCAACGCCTGTAAGAATCTCATCATGGACAATATTGAA

ATCGATAACGCACTTGGACACACTAAAGGTGGTCACAACACAGACGGTTTTGATGTGGCT

CATTCCGAGAATGTGAGAATCACCAACAGCCGAGTCAACAACCAAGATGACTGTTTGGCC

CTCAGCTCGGGTAAGAACATCGTGTTCGCCAACAACGAGTGCCGCGGAGGCCACGGGATT

GCCGTCATCGGCGGCTTCGACGGCGACGTAGCCGAAGACATCCTCATCAAAGACTGCAAA

GTTATCAACAATAACATCGGCGTCCGCGTCAAGACCATCCTCAACAGCAAAGGCTCCGTC

AAGAGGGTCACCTTCGACAACGTCGAACTCAAGGACGTCAGCGAAATAGGCGTCGTCGTC

ATCGGCAACTACTTCGGAAACAACGGCCCTAAAGGCGAGCCGACGAAAGGTTGTCCCATC

AATAACCTCATCATGAACAACATCCACGGGAACGTCCTTAGGAACGGAACCAAACACTGG

GTGTACGTGGCGGAGGGCTCTGACTGGGTCTGGAACACCAACATTCAAGGGGGCGAACGG

CCTTGGTTGCCTTGCAAGGGCATCCCTGCCGGCCTCAATATCGAATGCGGAACCAACAAG

GTTGGTTAA

>m_16624

ATGACCATGCTTTTCTTCACTTCTCCATTATTGAGCCTTCTACTAGTTGTAGGAGTGTCA

CTGGGTTTTGAACTTCAGAGGTTTGAACAACTGGATGATGCTAAACAACAAACATTCATC

AAGGTGAAAAACTTGAACGTTCCCGCAGGAAAGACGTTAGACTTGACTAAATTGAAAGAT

GGGACGACTATTGAATTTGTCGGGCGCACCACTTTTGGTTTCAAAGAGTGGGATGGACCC

TTGGTTAAAATTAGCGGAAAAAATTTGAAAATTGTTGGAGTGAAAGGAAACTTGTTGGAT

GCTGAGGGACAAAGGTGGTGGAATGGAAAAGGTGCCGAACGTGGTTTGAGAAAACCCAGA

ATGTTCGAGGCAATTGTAGACGACTCCATGATCACTGGTCTCAATTTTAAAAACCCACCT

CAAGCTTGCTTTGTATGTAACTGGTGTCACAATGTTCAAATCTCCTGGATAAACATTGAT

GCTAAAGATGGGAGAAATGGATTGGCTTTCAATACTGATGGGTTCGGTATCGGATATGCC

AAGAATGTCACGCTGACTGACAGTTATGTTTACAATCAAGACGATTGCTTCGTTACAGGA

GCCGGGGAGGATATTCTCGTTGATCGTCTCACTTGCGAGGGAGGTAACGGTATTTCAGTT

GGTTCCCTAGGTGGAGGTGCTAAGGTTGAAAGAGTTACGGTCAGAAACTCCAAAATCATC

AACAACTTGGTCGGTGTCAATGTGAAGACCGGATGGAACGTGAAAGGTTCACTGAAAGAC

ATCACGTTCGACAACATTGAGCTTGTCAACATTCAGCAATTCGGTATCAGCGTTCACGGT

AACGAAGGGCATCCCAACTTCCCTGCTGGTGATCCAACTCCGTTCCCTATTGAAAACTTG

ACCATCAACAACGTGAGAGGAAACGTAAACGGTGCTGGGGCTGCAAACACCTGGGTATGG

GTTGCTCCTGGTAGCGCTAAAAACTGGAAATGGAACTCCAATGTCACTGGTGGGAAGTCA

GCAATGTTCCGCCCACCTCTTCAATGCAAAGGAATTCCAGCTGGTTTGAAAATTCCTTGC

GCTGAGAAATAG

>m_19507

ACAGTCATCTTAGCAGAACACATTTCTCAAAAGAGGCTGGATACCACAATGATTTCACTT

GGGCTTTTGATGTTCTTGGCAGCAGCTTCTGCTGTGGATGTAAACGATATCAAGCAACTG

AATGCCGCCAAAAACTCTCAGCGCATTACTCTGCGAAACATCAACGTTCCAGCCGGAGTC

ACTTTGAATTTGGACAAACTCAAACCTGGGACTGTAGTTGAATTTGCTGGACAAATCACG

TTTGGGTACAAAGAATGGGAAGGGCCTCTTATCTTGATCGGCGGAAAGAACATCAAGGTT

GAAGGCAAACCAGGACATTTGATCAACTGCCAGGGAGAGCGTTGGTGGGACGGCAAAGGA

GGAAATGGAGGGAAGAAAAAGCCAAAGTTCATGGCTGTCAGGCTCACCGATTCGTCGATT

GACGGTCTCCAAGTCAAAAATCTACCAGCCCACGGATTTTCGGTTAACTCCTGCAAGAAC

GTGGCCATCTCCAGGATCAACTTGAACGTTGCTGATGGAGACAAGAAAGGAGGACACAAT

ACTGATGCATTTGATGTAGGTAACTCCGTAGGAATCAAAATCACTGACAGCTGGGTCCAC

AACCAAGATGACTGTTTGGCTATCAATTCTGGAACTGATATTACGTTCGAGCGCAACACT

TGCATTGGAGGACACGGAGTTTCTATTGGGTCTGTGGGAGGGAGGAAGAATAACGTCGTT

GAGAAGATTAGAGTCCGGCAGTGCAAAGTTATCGATTCCGACAACGGCATCCGGATCAAG

ACTGTAAAAAATACTTCTGGTTCTGTCACAGATGTATTGTTTGATGACGTGGAATTGAAG

AATATTGCCAAGCGTGGTATCGTCATCCAAGGCAACTACCTCAACAAGCGTCCAGAAGGT

GACCCTACAGGCGGAGTCCCCATCAAAGACCTGACCATCAACAACGTACGCGGTAACGTC

CTTCCAGCAGGAGTTAACGTTTACATTTGGGTTGCCAACGCCTCCAACTGGAAATGGAGT

AATATCAAAGTCGTAGGTGGAAAGAAAGATCTAGGACAGAAAGGAGTTCCTAATGGTGTT

AAATGGTAA

>m_24109

TTCTCTCCTAGCATCCATTGGTTTCAAGTGGACCTAGGTACCTATCTAACTGTGACATTC

ACGTCAACGAAAATGATGTCAAGCTTAGGCACCGTAGGGGGCCTTTTCTTGGTGATGGCT

CTCGCCTCTGCGGTAGATGTTAACAACATGCAACAGCTGGACGCTGCCAAGAAAGGTAAT

GACAAACGTATAGTCATCAGGAACCTTCAAGTCCCTGCTGGAGTCCAGTTGAACCTGGAG

AACCTTAAGCCTGGGACAGTGGTTGAGTTTGCTGGCCGTGTCACTTTCGGATATAAAGAA

TGGGACGGACCTCTCATCAAAATCTCTGGTAATAACGTCAGAGTCGAAGGAAAGCCTGGA

AATCTATTGGATGGTGAAGGCGCTCGTTGGTGGGACGGCAAAGGAGGCTCTGGTGGCAAG

AAGAAGCCCAATTTCCTTGAGTTGTTCAGGTTGGACAATTCGGTCGTAACAGGTTTGAAT

ATCAAAAACTCTCCTCTGAAGATTGTATTGATAAACTTCTGCAACCATTTGCAAATCAAT

AACATTAACTTGGACAATGCTGCTGGTAAAGGCAAAGCTTTCAACACTGACGGATTTTGT

GCTGGCGTTAACAAAGACATTAGAATCAATAACGTTAGAGTCCATAACCAAGATGATTGT

CTCTGCGTACTTGCAACTGACCAGATTTGGTTTGAAAATAGCGTCTGCACTGGTGGAAAT

GGAATTTCCATCGGATCCATGGGAGGTGGATACACAGTGAAGGGACTTACTGTTAGAAAA

GTACAAATCATTGATAGTTTCAATGGTTTGAGGATCAAGACCAAGAAAAATCAAAACGCC

TTGGTGCAAGATGTAACATGGGACGATGTTGTTCTTAAGGACATTCAACAGAGAGGTATC

ATCATCCACGGGAACTATCCCAACTGGCGCCCACAGGACGAACCTGACAACAAAATCCCT

ATTAGGAACCTTGTCATCAACAACGTTCGCGGAACTGTACAGAAAGGTGGCTCCAATATT

TGGATCTGGCTTGGCAATGGCGTCGCCTCAAACTGGAGGGTCAGCAATGTTAAGGTGACA

GGCGGTGGTCTTAAACTGGCTTGCAAGGGAATACCAAAAGGAGTCAACATCGCTTGTGGA

CAATAA

>m_24564

TCTTTTTCTCCATCGTCTTCGCCAGAGGTTGGAGATATCACGGCAAGGATGAACGCTGTC

CTAAGTTGTTTGTTGTTAGTGGTTGCAACCGCATCCGCGCAATACTTCGAATTGAAGAAC

GTCAATCAGTTGAACGAGGCGAAAAAATACCAGAAAATCGTCATTAGAGACCTCCAAGTT

CCAGCTGGTGTCACGTTGGACTTGTCCAACTTGAGAGAAGGAACGACAGTCGAGTTCGCC

GGACGTGTCACATTCGGTTACAAAGAATGGCGCGGACCTCTAGTCAAGATCAGCGGAAAG

AGGTTGAATATTATGGCTTACGATTACGCAAGATTGGATGGTGAGGGCCACAGGTGGTGG

AAGGGCGGCCGTCTCTCTAAACTGGTGAAGCCTAGGTTCTTCGAAGCTACCGTTGACGAT

TCTACGATTCGTGGACTGTACTTCAAGAATCCTCCTGCATGGTGCTTCGTTTGCAACTGG

TGCCACAACACTGAGATTTCCCGTATGACAGTTGACACCAAAGATGCCGGAGATGGCAGG

GCTGGACGTGCTTACAACACTGATGGTATTGGTTTGGGTTACGTCAAGAACGTGACGGTT

CTCAACAGCTACGTGTTCAACCAAGACGACTGTTTCGTGACTGGAGGCGGTGAAGACATC

CTTGTGGATAACTTGACCTGCGAAGGAGGTAACGGAATCGGAGTCGGATCTCTTGGAAAT

GGCGCTGACGTCAAGCGTTTGACCATCAGAAACAGCAGAGTTATCAACAGTTTGACGGGA

CTGAACATCAAGACTGAGACAAACGCTGTTGGTCTCCACAGGGATGTCACCTTCGACAAC

ATCGAACTCAAAGATATTCACCAGTATGGAATCACCATTCACGGAAACGAACTTTCCCCT

ACTTTCCCCCGTGGTGAGCCAACTCTCTTCGCCCTCGAGAACTTGACCATGAGAAACATC

AGAGGAAACATGGTTGGACCTGGAGGTGCCAACGTTTGGATTTGGCTCCATCCCAACAGC

GCTAGGAACTGGAAGTGGCAAAATGTCAACGTTAGA

>m_24565

TCTTTTTCTCCATCGTCTTCGCCAGAGGTTGGAGATATCACGGCAAGGATGAATATTGTT

CTTGGTTGTTTATTATTCGTGGTTGCAACCGCATCCGCAAAAACTTTTGAATTGAATAAT

GTCAACCAATTGAATCAAGCGAAAAAAATGATGAGAATTATCATTAGAAACCTCCATGTC

CCAGCTGGTGTCACATTGGACTTGACCAATTTGAAAGATGGAACCACGGTCGAATTTGAC

GGACGCGTCACATTCGGTTACAAAGAATGGCGCGGACCTCTAGTCAAAATAAGTGGAAAG

AAGTTAAAAATTATAGGTCATCCTCATGCTAGATTGGACGGCGAGGGCCAAAGGTATTGG

AAGGGTGGTCGTAACACTAAAATGTTGAAACCTAGGTTCTTCGAAGCTATAGTTGACGAT

TCTACGATTCGTGGCCTGTACTTCAAGAACCCTCCTGCACCTTGCTTCCTTTGCAACTGG

TGCCACAACGTCGAAATTTCCCAGATAACAGTTGACGCCAAAGATGGCGGAGACGGCAGA

GCTGGACGTGCTTTCAACACTGATGGTATTAGTTTGGGTTACGTCAGGAACGTTAGAGTT

CTCAACAGCTACGTCTTTAACCAAGACGACTGTTTCGTTACTGGGGGCGGTGAAGACATG

CTTGTCGATAACTTGACCTGCGAAGGAGGTAACGGAATCGGAGTTGGATCTCTTGGAAAA

GGCGCTGACGTCAAGCGTTTGACCATCAGAAACAGCAGAGTTATCAACAGTTTGACGGGA

CTGAACATCAAGACTGAGACGGGCGCTGTTGGTCTCCACAGGGATGTCACCTTCGACAAC

ATCGAACTCACAAACATTCACCAGTATGGAATCAGCATTCACGGAAACGAAGGTCCTACT

TTCCCCTTTGGTGAGCCATCTTTCTTCGTCCTCGAGAACTTGACCATGAGAAACATCAGA

GGAAACATGGTTGGAACTGGAGGTGCCAACGTCTGGATTTGGCTTCATCCCGCCAGCGCT

AGAAACTGGAAGTGGCAAAATGTCAACGTTAGAGGAGGCAAGAGTGCGATGTGGAGGCCA

CCACTTCAGTGCAAGGGAGTTCCTCC

>m_24566

TCTTTTTCTCCATCGTCTTCGCCAGAGGTTGGAGATATCACGGCAAGGATGAACGCTGTC

CTAAGTTGTTTGTTGTTAGTGGTTGCAACCGCATCCGCGCAATACTTCGAATTGAAGAAC

GTCAATCAGTTGAACGAGGCGAAAAAATACCAGAAAATCGTCATTAGAGACCTCCAAGTT

CCAGCTGGTGTCACGTTGGACTTGTCCAACTTGAGAGAAGGAACGACAGTCGAGTTCGCC

GGACGTGTCACATTCGGTTACAAAGAATGGCGCGGACCTCTAGTCAAGATCAGCGGAAAG

AGGTTGAATATTATGGCTTACGATTACGCAAGATTGGATGGTGAGGGCCACAGGTGGTGG

AAGGGCGGCCGTCTCTCTAAACTGGTGAAGCCTAGGTTCTTCGAAGCTACCGTTGACGAT

TCTACGATTCGTGGACTGTACTTCAAGAATCCTCCTGCATGGTGCTTCGTTTGCAACTGG

TGCCACAACACTGAGATTTCCCGTATGACAGTTGACACCAAAGATGCCGGAGATGGCAGG

GCTGGACGTGCTTACAACACTGATGGTATTGGTTTGGGTTACGTCAAGAACGTGACGGTT

CTCAACAGCTACGTGTTCAACCAAGACGACTGTTTCGTGACTGGAGGCGGTGAAGACATC

CTTGTGGATAACTTGACCTGCGAAGGAGGTAACGGAATCGGAGTCGGATCTCTTGGAAAT

GGCGCTGACGTCAAGCGTTTGACCATCAGAAACAGCAGAGTTATCAACAGTTTGACGGGA

CTGAACATCAAGACTGAGACAAACGCTGTTGGTCTCCACAGGGATGTCACCTTCGACAAC

ATCGAACTCAAAGATATTCACCAGTATGGAATCACCATTCACGGAAACGAACTTTCCCCT

ACTTTCCCCCGTGGTGAGCCAACTCTCTTCGCCCTCGAGAACTTGACCATGAGAAACATC

AGAGGAAACATGGTTGGACCTGGAGGTGCCAACGTTTGGATTTGGCTCCATCCCAACAGC

GCTAGGAACTGGAAGTGGCAAAATGTCAACATTAGAGGAGGCAGGAGTGCGATGTGGAAG

CCACCACTTCAGTGCAAGGGAGTTCCTCCCGTTGGAATCCGTTGTGCTGAGAAGTAA

>m_24567

TCTTTTTCTCCATCGTCTTCGCCAGAGGTTGGAGATATCACGGCAAGGATGAACGCTGTC

CTAGGATGTTTGCTGATTGTGGTTGCAACTGCATCTGCGCAATATTTTGAATTGAGAAAC

GTCAACCAACTGAATGAAGCGAAAAAATTCCAGAAAATTGTTATTAGAGACCTCCAAGTC

CCAGCTGGTGTCACATTGGACTTGACCAATTTGAAAGATGGAACCACGGTCGAATTCGCC

GGACGCGTCACATTCGGTTACAAAGAATGGAAAGGACCTCTGGTCAAGATAAGTGGAAAG

AGGTTAAACATTATAGCTCAACCTCAAGCTAGATTGGACGGCGAGGGCCACAGGTGGTGG

AAGGGTGGTCGTAATACTAAAATGTTGAAACCTAGGTTCTTCGAAGCTATAGTTGACGAT

TCTACGATTCGTGGCCTGTACTTCAAGAATCCTCCTGCATGG

>m_2549

ATGAAATATTTCTTCATCGCTGTATTTGTGTCTGTGGCCTCAGCAGCTGACATATGGAAT

CTCCAGCAGTTAGAGGCTGCCAAGAAAGCTAAAGACAAAAACATCGTCCTAAGAGACATT

CAAGTTCCAGCAGGTCAAACTCTGGAACTTCAGGGTCTGGAAAATGGTACCAGTATCACA

TTTGTTGGACGAATCACATTCGGATACAAAGAGTGGAAGGGACCGTTGGTGATCATCAAA

GGGCATAACTTCCATGTGGAAGGTAAGCCAGGACATGTAATAGACGGTGAGGGACACCGC

TGGTGGGATGGTTTAGGAGGCAATGGCGGCAAAATCAAACCTTACGGAATCTATGTTCAG

CTAACGCATTCCAAGGTTAGAAATATCAAAGTAAAGAATTCACCCAAACACTGCTGGGCT

ATCAACGCTTGCCGTCACGTAGTGTTTGACGGGATAATTGTCGATGATACTGACGGTCAC

GCTAAGGGAGGGCACAATACTGATGGATTCGACATTGCCAAATCTCACCATGTGAAGATA

AAGAACAGCTGGGTCAACAATCAGGATGACTGTTTAGCTTTGAACTCAGGAACTTTCATA

ACGTTTGAGAACAACACCTGTGAAGGAGGGCACGGCATTGCTGTAGCTGTTGGGGGTTAT

GACGAAAACGTCGCCAAACACGTTTATATCAGAAACTGCAAAGTCATTAAGAACAATATC

GGTATCAGAGTGAAGACTCTGTTGAACGGCAAAGGTATTGTAAAGGATATCAATTTCGAG

AATGTGGAGCTTAAGGACATCAGCCAAACCGGGATCGTCATCATCGGCAACTATCTGAAC

TCAGGGCCGAGGGGGGAACCCACAGGAGACTGTCCCATCCAAGATCTGAAAATTGACAAT

GTTCGAGGGAACGTACTCAGGAACGGAACTAACATTCAGGTCTGGGTAAAGAACGCTTCA

AACTGGAAATGGAAATCTCAAGTTGTGGGTGGTACGAAGAAAATACCCTGCCAAGGTGTC

CCTAAAGGGGTAAACATACAATGCGGTTAA

>m_27405

ATGAAATTCGTTCTCTTCGCATTGGGTGCGATCGTTGCTGTGGCATCAGCCGTTGATGTT

CACAATCTGGAGCAGCTCGAGGCCGCTAAGAAAGCCAAAGACAAGAACATTGTGCTGAAG

AATATCCAAGTCCCAGCCGGGAAAACACTGGAACTCCAAGGTCTGGAGCCTGGAACCAAA

GTCACCTTCACTGGACGTATCACTTTTGGATACAAAGAATGGAAAGGACCTCTCGTGATC

ATCAAAGGACACAAGCTGACTATTGAAGGAAAACCTGGACACTTGATCGATGGAGAGGGA

CACCGTTGGTGGGATGTTCTTGGAGGAAATGGCGGAAAAGTCAAACCCTATGGTATCTAC

GTTCAGCTCACTAATTCCGTTGTCAATGGACTCACCGTGAAGAACTCTCCTAAACATTGT

TTCGCCATCAATGCTTGCGAGAACACCGACTTTATTGGAATCACGGTCGACAATGCTGAT

GGACACAAAAAAGGAGGCCACAACACTGACGGATTTGATGTAGCCAAATCCCATAACATC

AAGATCATTAACAGCAAAGTAAACAATCAAGACGACTGCTTGGCCATTAACTCAGGTACC

AACATTCTCTTCCAGAACAACATCTGCGAAGGAGGTCATGGTATTGCTGTTGCCGTCGGT

GGCTATGATGTCAACGAAGCCAAGAACATTGTCATCAAGGACTGTCAAGTTATCAAGAAC

AATATCGGTATCCGAGTGAAAACTCTGCTCAATGGTAAAGGTATTGTTGATGGGGTCACT

TTTGACAACGTTGTTCTTAAAGACATCAGTGAAGTCGGTATTGTCATCATTGGAAACTAC

CTCAACTCCGGACCCAGAGGTGACCCCACCGGCGACCTCCCCATCCGTGGACTGACCATC

AACAACGTCCGTGGAAACGTCCTGAACAACGGAACCAACATCCATGTCTGGGTGAAGAAC

GCTGCCAACTGGAAATGGAACTCCAATGTCGTGGGAGGAACAAGGAAGAAGGAATGCAAG

GGAGTTCCCAACGGCGTTAAAATACAATGCGGTTAA

>m_28554

ATGGCTGTCAGACTTAAAGAATCACCGATTGATGGACTGAATATTAAAAATATACCAGCT

CACGGATTTTCAATCAACTTGTGTAAGGACGTGGATATCACCAGGATACACCTAGACGTT

GGTGAAGGGAAATGGAGAGGAGGGAATAACACAAATGGATTCGATGATGGTAAGTCAAAA

GGAACCCGTATCGCTTACATCACTGTCATCAACCAAGATGACTGTTTAGCGATCAATTCT

GAAGCTGACATCAGTTTCGAGAACAATGTTTGTGTCTCACGATTTACGCTACAACTTGAT

TTACTTCTTCCACTCAACCGTAGAAAATCTTCTAATTTTCATATAGATTCATGTTATCGA

TACAGAGAAGCTATGTGCAACACCCACTTTTTTTCCAACCGGACTATCTTTGATTACCAA

TGGTCATTCAGTCGAGTTTTGTCTCACAGAAGCAAGTTGTACGCTCTGATGAAAGTCATC

GTTCACAGATCAATACTAACCTTTGCGCATGCGCAAACTTAA

>m_29450

TCCTCAGGGCCAATCAAGACTGGAATGCTTGTGAACATGGCCCTCACATATATAGCAGGA

AGTTTACTGCTCTCCATCGTAGCAGTCACTGCTGTGGACGTGTGGAACATCCAACAACTG

GAAGCTGCCAAGAAAGGAAGCGACAGCCAAATATTTATCAGAAATTTGGAGGTTCCTGCC

GGTACCACACTCAACCTGGAAAATCTAAAACCTGGGACGTACGTGAAATTCGTGGGCGTC

GTTACTTTTGGTTACAAGGAATGGGTTGGTCCCCTTATAAAGATATCTGGAAAAAACATT

AAAGTAGAAGGGACTAGGGAAAGCTTAATTGATGGCAATGGAGCTCGCTGGTGGGATGGC

AAGGGGGGAAATGGAGGCAAAAAGAAGCCTAAACTACTATCGTTGTGCTTAACAGACTCA

GAGGTCACTAATTTGAATATCAAAAACTCTCCAGCTCATGGTATTTCTGTAAATTGTAAG

AACGTCAATATCTACAATATAAACTTTGACAACAAAGATGGCCACTCTAAAGGTGGGCAT

AATACTGATGCGTTTGACGTCGGTAATTCAGATCGTGTCACCATAGCTAATTGTCATGTG

GAAAACCAAGATGATTGCTTGGCTGTCAATTCTGGAACCCGGATCGTATTTGAGAAGAAC

ACCTGCATCGGTGGGCATGGCATTTCCATAGGATCCGTTGGCGGGAGGAGCAACAATGTC

GTGGATGATGTTATTGTCAGGGATTGCAAGGTCATTAACAACGACAACGGTATCCGCATC

AAGACCAACAAGGATACAACCGGGCTCGTCAAGAACGTCCGTTTCATCAACGTTGAGCTT

CAGAACATCGGAAAAGTCGGGATATCCATTCAAGGGAACTACGCCAACTCCGGCGCCAAG

GGTGACCCAACGGGTGGCGTACCCATACAGGATTTGCTGATAGACAACGTCTATGGGACT

GTGAGTCCCAAAGGCGTCAACACATTTGTCTGGGTAGCCAATGCATCAAGATGGACTTGG

AAATCCAACGTCAAAGGCGGCAAGAAAAAGGTCGATTGCAAAGGGATCCCAGCAGATTTG

AAAATTCCTTGCGGCATTTAG

>m_30050

ACAGCTACCTGTAGGTCGACGGTTGGGATAAAAATTGCCTCTGCCAGTATGACATCCATC

GCTGCCTCCTTCGGAGGCCTCCTTCTCGTCCTGGCAGTGTCGTCAGCCTTCGATCTCAAC

ACCTTCGATCAGCTTGATGCTGCCAAGAAAAGCGCCGACAAGCTCATTGTTATCAGAAAC

CTGGTAGTTCCAGCTGGTCAGAAGTTGGACCTGACGAATTTACAACAAGGAACCGTTATC

CGATTCGCCGGCCGTGTGACGTTCGGTTACCAAGAATGGGACGGTACCATGATTCAAGTC

AAAGGAAAGAACATCAGGGTTGAAGGCAAGCCAGGAAATTTGATCGATGGTGAGGGTCAC

CGTTGGTGGGATAAGAAAGGAGGGAATGGTGGAAAAAAGAAGCCTCGATTCATGGAAGTG

AATCTAGAAGATTCTATTGTGACTGGTTTGAACATCAAAAATCCTCCAAGACATTGTTTT

GTTGCGAACTACTGCAAAAATGTTCGTATCGAGTATGTCAACATTGACATTAAAGAAGGT

GACACAAGGGGAGCCCATAACACTGATGGTTTCGGAGTCGGTGGATCCCAGAACGTGACG

GTAGCTAACTGTAAAGTTCACAACCAGGATGATTGTTTCTGCACTGGATCTGGGAGCGAT

ACAGTTTTTGAAAACAATGTCTGCACTGGTGGACATGGAATTTCCATTGGTTCGATGGGT

AACGGACAAAAAGTAGAAAGAGTTCACGTCAGAAACTGTCAAATCATCAAAAACACCAAC

GGAATCAGGATTAAATCCAGAAAGGGAGAAACCGGACTCGTACGTGACGTCACATTCGAG

AACATTGAGATGAAGGACATCACTAAGTATGGAATCATCATTCAAGGGAACTACTTGAAC

GGCGGTCCAAAAGGTGACCCTACTCCTTTCCCAATGGAAAATATTGTCATCAAAAACGTG

AGAGGAACTGTCAGCAGGAAAGGTACGAATATTTTGGTCTGGGTTGCTCCTGGAAGTGCC

AAAAACTGGCAATGGAATTCCAACATCACTGGGGGGCAGAGAGAAGTCTCTTGCAAAGGC

ATACCCCAAGGACTTAACATCCCCTGTGGTAAAAAATGA

>m_47023

ATGACCGAGTCATTAATGAAAAAAAATATGAAGCCATTACTCAATACACTGGGTATCTTC

TTCCTCCTTGTGGCCGTAACAAATGGATTTGAGCTCAAGAGTTTTGACCAACTAGACGCA

GCTAAAAAAAGTTCTGACAAGCATATTGTTATTCGCGATCTCTTCGTGCCTGAAGGCAAA

GTACTGGACTTGTCTAAGTTACAGGATGGAACTCTCGTTGAATTCGTGGGACGGGTGACA

TTTGGTTTTAAGGAATGGGATGGCTTCATGGTTTTGATAACAGGAAAGAACATTAGGGTA

GTTGGCAAGCCTGGACACTTAATCGATGGCGAAGGCCATAGATGGTGGGACGGCAAAGGA

GGTAGTGGAGGCAAGAGGAAGCCAAGATTCATGCAGGTTACGTTGGAAAACTCTTTAGTA

TCAGGATTGAACATTAAAAATACTCCAAAAGACGCCTTCGTCGCTAATTTCTGTAAGAAT

GTGCGCATTGAGTATTTGAACGTTGATATCAAGGATGGTGATAGAAAAGGGGGCCACAAT

ACTGATGGAATCGGTGTTGGAGGATCGAGTAATGTCACAGTTTCAAACTGCAAAGTCCAT

AATCAAGATGACTGTTTCTGCATTGGTTCTGGAAGTGATACTGTTTTTGAAAATAATGTT

TGCACTGGTGGGCATGGAATTTCCATCGGGTCTATGGGCGGCGGTAAGAAAGTCGAACGA

CTTCTCGTCAGGAACTGTACTGTCATCAAGAACACCAACGGCATAAGGATCAAGTCGAGA

AAAGGTGAGACCGGTCTGGTCAAAGATGTCACGTTTGAAAACATCGAGCTTAAAGACGTA

ACGCAGTACGGCATCATTATTCACGGAAATTACCCGGACAATGGTCCCAAGAGTGAACCA

ACTCCTTTCCCTATTGAAAATTTGACTATCAACAACGTACGAGGGACTGTCGGCAGAAAA

GCAACCAACATTCTTGTCTGGATCGCTCCAGGAAGCGCCACGAACTGGAAATGGACTTCT

AACGTTACTGGTGGAAAGAGGAGACTTTCGTGCAAAGGAGTACCAGCAGGAATCAATATG

CCATGTGGAAAAGTGTGA

>m_47531

GATTCATCGTTATCCAATCTCAGGATTTCTCCGATTCCACGAGACATCCAGGTCGAAATG

ATGATGTTTAGTCCTTCAGCTGTCGTAGGCTTTATGGTGGTGACGGTTGCTCTAGCAGTC

AATGTGGACAGATTTGACCAAATCGAAGCAGCAAAAAAAAGTAACGATAAAGTAATCGTC

ATCAGAAACCTGCAAGTTCCTGCTGGGGTGTTGCTGGATTTGCAAAACCTGAAACCTGGT

ACCACCCTTCAGTTCCAAGGGCGCGTCACGTTTGGTTACAAAGAATGGAAAGGACCTATG

GTGAGAATATCGGGGAAGAACATCATTGTTGAGGGTAAACCTGGACACGTTATAGATGGT

GAAGGCGCCCGTTGGTGGGACGGACTTGGAGGAGCTGGTGGAAAAACGAAACCCACATTC

ATCGAACTAAAATTGGATGATTCAATCGTAAGAGATTTGCACGTTAAAAACACACCAGTA

CAGATGTTCAAAGCCAGCTTGTGTAATAATCTCCTGATCACCAATGTGAACCTTGATAAT

GAAGACGGCAAGAACGGTAAGGGCCGCAACACTGATGGATTCGCAATGGGACTTTCCAAA

AACGTCACTGTACAAAACAGCCGTGTCTATAATCAAGACGATTGTTTCTGCATCGGCGGT

GGAAGTGACATTAAATTCATAAACAACGTCTGTATTGGAGGGAATGGAATCTCCATTGGT

TCTATGGGAAATAACCGAGTGGTTGAAAGAGTCGAAGCCAGACATTGCCAAATCATCGAC

AGCTTCAACGGTATTCGAATCAAAACGAGAAAGAACGAAAAAGCGCTGGTCAAAGACGTG

ACATTCGATGACATTGTTCTGAAAGATATCCAACATAGGGGTATTATTGTTCATGGAAAT

TATCCATCATGGCGCCCAACTGATGAGCCAACAAACGGTTGTCCAATCCAAAACTTGGTG

ATCAACAATATTCGTGGAACAGTCATACCTGGTGGAGCCAACACCTGGATCTGGCTCGCT

AAAGGAGTCGCGTCAGGGTGGAAGGTCAGCAACGTGAATGTTACCGGCGGGAAATTGAAG

CTTGAATGCAAAGGACTTCCTGGCGGCATTGACAAAAACTTCCAGCAAAGATGTGGTCAG

ATTTAA

>m_50085

ATGGGACCGATGAAGCCGTCTCTCGGATGTGCTTTTCTGTTAATCGCTGTATCATACGGT

TTCGATCTCAACAACTTCGATCAGCTGGACGCCGCCAAAAGAAGCCCCGATAAACGTATC

ATCATCAGAGATCTATTCGTACCGGCAGGCAAGACGTTGGATTTGACAGAATTACAGCCA

GGAACCGTCATTGAGTTTTCAGGTCGAGTCACTTTCGGGTATCAGGAATGGGACGGTTTT

ATGGTTAGGTTGAAAGGGAGGGACATCAGGGTGGAAGGCAAACCTGGGAACCTGTTGGAC

GGTGAAGGACATCGCTGGTGGGATGGAAAAGGAGGTAATGGTGGGAAAAGGAAGCCTCGA

TTCATGCAAGTGACTCTGGATGATTCTATTGTAACTGGATTAAACATAAAAAACACTCCC

AAAGACTGTTTTATAGTGAATTGGAGCCATAACCTTCGTGTGGAGCGTATCAACATTGAC

ATTAAAGACGGAGACACGAAAGGTGGACACAATACTGATGGTTTTGGGGTTAGTGGTTCA

AAAAACGTAGTAGTAACAGATTGCCAGGTTCATAACCAGGACGACTGCTTTGCTACAACT

TCTGGGAGCGACACGATCTTCGAAAACAGCAAGTGTACGGGCGGGCATGGAATTTCCATC

GGGTCT

>m_50368

AGTCTGAACAGTGTAATTTATTCCTTTGTGGTGACTACAACGACAAAACTGATAATTGGT

GACATCATGAATTTGCTTTATTCCATCGGTGGTTTAATAGTGATTGCCGCTGTTACTGCT

GGATTCGATCTCAACAACTTTACCGACTTGGACGCAGCAAAGAAGAGTTCTGACAAACGT

ATAGTTATAAAAAACTTGCTAGTTCCGGCTGGTAAAACACTAGACTTAACCGGATTACAA

ACTGGTACAGTTATTGAGTTTACAGGGCATGTGACGTTCGGTTATGAAGAGTGGGACGGG

GAAATGATAAAATTGAAAGGGAAAAACATAACAGTGGTTGGTAAGCCTGGGCATCTTTTG

AACGGCGAAGGAAAGCGTTGGTGGGATGGAAAAGGTGGAAACGGTGGCAAGAAAAAACCT

AGATTCATGCATGTGTCTCTAACTGATTCTACGATTACTGGATTGCATATAAAAAATACT

CCACGGCATTGTTTCATGATCAACTCAAGTCAAAATCTGCGAGTGCAAAATACTACAATC

GATATCAAAGATGGTGCCAAGCACGGAGGGCACAATACTGATGGTTTTGGAGTCAGCAGT

TCGCGCAATGTGACCATCTCTAATTCTGTGGTTTACAATCAAGACGACTGCTTTGCAACG

ACATCTGGCAGTGACACAGTTTTTGAAAACGCAAAATGCGTTGGCGGTCATGGGATTTCT

ATTGGTTCTATGGGTTCTGGAAAAGTTGTGGAAAGAGTGATGATAAGGCATTGCCGCGTA

TTGGCCAATTCCAATGGGATACGCATTAAAACTAGGAGAGGAGAAACCGGAGCAGTTAAA

AACGTTACGTTCCGAGATATAGAAATGAAAGACATATCCAAATACGGAATTGTTATTCAG

GGAAACTACTTCAACAGCGGGCCCAAAGGAGACCCCACGCCTTTCCCAGTTGAAAATCTG

GTGATTGATGATGTTCGAGGTCACGTCATGAAATCGGGTGTGAATATTTTGGTCTGGGTA

GCTCCGGGAAGTGCTAAAAATTGGACTTGGAGTTCCAAAATCACTGGGGGACATAAAGAG

CAGGAATGCAAAGGTGTACCAATGAATCTAGGCATCCGGTGCGGGAAGAAATGA

>m_50380

ATGGCATCGAAACTCGTGACCTTTACAGGCCTGGTGCTGTGCATCACAGTCGCCGCAGCA

GTGGACATCTGGAGCGTCGATCACCTCGAACAGGCAAAGAAAGGCAATGACCCAGTAATC

AGAGTGCGAGACATCAACGTTCCAGCTGGTCGCCCTCTAGATTTCCAAGGTTTAGATGGC

AGAACTATTGAATTCCACGGGCGAGTTACCTTCGGTTACAAGGAATGGCAAGGTCATTTG

ATCATCATCAAGGGTAAGAATATTAAAGTTAAGGGTATGCCAGGCCACTTGATTGATGGT

GAAGGTCACCGCTGGTGGGACAAGTGTGGAGGTAACTGCGGCAAGAAGAAGCCTTTCTTG

ATTTACACTCAGCTTCAGGACTCCACAGTCGATGGGCTTAGGATCAAGAACACTCCTGCC

TGGTGCTTTGCCATCAACGAATGCAACAATGTTCACTACTCCAACATCGATATTGACAAC

AAAGACGGTCATACCAAAGGAGGCCACAACACTGATGGATTCGATGTCCACAAGAGCAGA

AACATCAGGATTTACAATAGCAAGGTCAACAATCAAGACGACTGTTTGGCCATCAACTCT

GGATGGGACATTGTCTTTGAAAACAACGTCTGCGAGGGAGGACATGGTATCGCTGTTGCT

GTTGGTGGTTATGATGTCAACGAAGCTAAGAACATCTTGATCAAAAACTGCAAAGTTATC

AAAAACAACATTGGAGTCCGCGTCAAAACTTTGTTGAACGGCAAAGGTATTGTTGACGGA

GTTACTTTCGACAACGTTGAATTGAAAGACATTTCTGAGATCGGAATCGTTATAATTGGA

AACTACTTGAATTCTGGCCCACGTGGTGACCCCACTGGAGGAATCCCAATCAAAAACTTG

AACATCAATAACGTGCGTGGTAATGTTCTCCACAACGGAACCAACATTCAGATCAACGTC

GCCCCTGGCAGCCCATCGGGATGGATCTGGAAGTCCAACGTTTGGGGAGGAAGGAAGAAT

CCCAACTGCAAAGGAACTCCTGGAAACCTCAACAACCCATGCAACTGGTAA

>m_50432

ATGAGAGCGAACGTGGTGACCTTCGGAGTCGTCCTGGTGGTCTTGGCGGCTGCCCACGGG

GCCGTGGTGACGGACTACAACCAATTGGCTGCTGCTAAACAGGGCAACCACATCACGCTG

CGAAACCTGCAGGTCCCAGCTGGCGTTACTTTGGACCTGACGAAGCTCAACCCCGGGACA

ACCGTCGAGTTCGATGGCCGTACGACTTTTGGCTACAAAGAGTGGGCCGGTCCTCTGGTG

AAAGTCAGCGGAAAGAATTTGAGGATCGTCGGTCTCCCTGGGAACCTCCTAGACGGCGAA

GGGAAACGCTGGTGGGACAAACTCGGAGGGAACGGTGGAAAAACGAAACCAAGGTTCATG

GAAGTCAATATTGATGATTCTTCTATTACTGGTTTGAACATCAAAAACCCTCCTGCATGG

TGTTTTGTGGCCAATTACTGTAAAAACGTTCACATCTCAAACGTTAACATCGACATCAAG

GACGGTGATAAGCAGGGAGGCCACAACACTGACGGGTTCGGCGTCGGGTACAGCAAAAAT

GTGACTATCCAAAACTGCAAGGTCCACAATCAGGATGATTGCTTCGTCACTGGAGCTGGC

AGTGATATCGTCATCGACAATCTATCCTGCACTGGAGGTCACGGCATTTCAATTGGATCT

TTGGGCCGTGGAGCGGTTGTGGAAAGAGTTTTAGTCAAGAACAGTAAAGTTGCCAGAAAC

ATGGTCGGGATCCGGATCAAATCCACCAGAGGTGAGACTGGAGCTATCAGAGACATCACG

TTTGACAACGTCGAGCTTCAAGGAATCACAAGATACGGTATTATAATCGAAGGGAACTAC

CTGAACTCTGGTTCAGCTGGTGACGCCACTCCGTTCCCAATTGAGAACATCACCATCAAC

AACGTCCGAGGCAGTGTTGTACGCAAGGCCACTAACATCTACGTCAACATCCATCCCACT

AGTGGTAAGAATTGGAAATGGAACTCAAACGTGACCGGAGGGCAGAAAGAACTCAAGTGC

ATTGGTGTTCCTGCTGGTCTCAATATCCCTTGTGGTAAGAAACAGTAA

>m_51390

ATGGTTCCTTCAATTTGTGGGCTTTTCGTGCTGGTCGCTGCTGCTTCAGCTGTTGACGTG

TGGAACCTGCAGCAGCTGGAAGCTGCCAAGAGGGGAAATGATCTCACCATAAACGTCAGG

GACATTTTCGTACCAGCCGGCCAGACCCTAAACTTCGAGTTTGTGAAGCCTGGAACCACT

ATTGTGTTCAGAGGACGAGTCACTTTCGGCTATAAAGAATGGAGAGGACCTCTCATTATT

CTGAAGGGAAAGAACCTCAAGATCAAAGGAGCAGATAGGCACATCTTCGACGGTGAAGGT

CGCCGTTGGTGGGACGGAACTGGCACCAACAGTGGTAAGGTGAAGCCGTACATGTTTTAC

GTTCAACTGACAGATTCAAGCGTAAGAGGTTTGAACGTAAAAAACTCTCCTGCTCACACA

TTCGCCATCAACGACTGCCATCATATCTCAGTCAACAACGTCATGATTGACAACAGAGAC

GGCGACAGGTTCGGAGGCCACAATACTGATGGGTTTGACATTGCTAAATCCGACCGCGTT

CTCATCGCCAACAGCACGATTTACAACCAGGATGATTGTTTGGCTATCAACTCCGGTAAT

GACATCACTTTCCAGAGAAACAAGTGTATTGGAGGGCACGGAATGGCCATCGCGGTTGGA

GGATACGATGTTAACCAAGCAACAAACATCAGGATTCGAGGTTGTCGCGCCATAAAAACC

AAATACGGAGTTCGCATCAAGACTCTAAGGGGAGGTCGTGGATTGGTTAGAGGAATCAAC

ATCGAAAACATCCTTCTCAAAGACGTCACTGACGCTGGACTCCTGATTATCGGCAACTAT

CTTAACTCTGGGCCGGGAGGTGAACCAACCGGAGGCATCCCAATCCAGGACTTGCGAGTG

GACAACGTTCGTGGAAATGTCCTAAGCAAAGGAACCAATATACACGTTTTCGTCGCCAAT

GCCAGGAACTGGAGTTGGAATTCCAACATCCAAGGAGGCCAAAGGAGACTGCCTTGCAAG

GGAATTCCTAACGGTCTTCGCATCCCCTGCGGTTAA
