# Supplementary material for: Duplication and expression of horizontally transferred polygalacturonase genes is associated with host range expansion of mirid bugs
Source: BMC Evol Biol. 2019 Jan 9;19:12. doi: 10.1186/s12862-019-1351-1 (PMC6327464; doi:10.1186/s12862-019-1351-1)
Supplement: Supplementary file 3 — The coding sequence of PGs identified in this study. (ZIP 71 kb) [file 12862_2019_1351_MOESM3_ESM.zip › Additional file 3-The coding sequence of polygalacturonase genes in Adelphocoris lineolatus.docx]

>m_11741

ATGCAGCCGTGGTTCCTCTTAATTACGCTCCCGTTGATGAGCGGAATAATTTCAGCTTCA

GAGTATACAATTAGAAGCATCAGCGATGTAAAATACGCCAATAAATACAGCGATATAACA

ATTTCGAATCTCCATGTGCCAGCCGGAGTTCCACTGGTGTTAGTGGGGTTGAAGAATGCA

CGTGTCACATTTGAAGGGACCACCACGTTCGGTTATAAAGAGTGGAAAGGGCATTTGATG

ATGTTCAAAGGGGACAACGTCACCATAACTGGGTCTCCTGGACATTTGATAAACTGCGAG

GGAGAGCGATGGTGGGACGGACTGGGAGGTCTCGGCGGAACAAAGAAACCCAAGTTCTTT

GAAGTCCGCCTCAACAATTCGCAAATCTTCGGACTCCAAATCAAGAACACTCCAATGCAC

GCGATTTCCATCAACCACTGCAACAACTTAGTGGTGTCGGACATCATCGTGGACAATCTG

GAAGGCGACTGGAAAGGTGGTCACAATACAGATGGATTCAACGTGTACGAATCAAAAAAC

GTCACCATCAGAAACTGTAATGTACACAATCAAGATGACTGCATAGCCGTGAAATCTGGG

ATCGACCTGCTTTTCGAGAACCACTTCTGTTCAGGGGGCCATGGGATTTCAATTGGTTCT

GTAGGAGGTCGTAGGGACAATATCGTTGAACGGGCTACTATACGAAATTGCGTCATTCAG

AATTCTGTCAACGGTGTTAGAGTGAAGACAGTCAGGAAGGCTATTGGAAGGGTGTCAAAT

GTGACCTTCGAGAATGTTGCATTGTCGAACATATCCGAATTCGGTATCACAATGCAAGGC

AACTACAACCTGGAACACGGAGATCCGAAAGGCGAACCTACAGGAGGGGTCCCGATCAGT

GGACTCGTCATAAACAACGTGCGCGGCACGGTGAACCCAAAAGGAACCAACGTCTGGGTC

TACGTGAAGAACGCCTCAAATTGGAGTTGGGACTTCCACGTAACGGGAGGAACCAAGACT

AGGCGTTGCGAAGGTTTCCCAGATGGAATGACTCCAGTATGTTGA

>m_26006

TCTTCTTCGGTTTCTTCACCAGACGTTGGGGAATTCACGGCAAGGATGAACGCTGTGCTA

AGTTGTTTGCTGTTTGTGGCTGCAACCGCATCCGCACAATATTTCGAATTGAAGAACGTG

AATCAGTTGAACGAGGCGAAAAAATACCAGAAAATCGTCATTAGAGACCTCCAAGTTCCA

GCTGGTGTAACGTTGGACTTGTCCAACTTGAGAGAAGGAACCACGGTTGAGTTCGTCGGA

CGAGTCACATTCGGTTACAAAGAATGGCGTGGACCTCTGGTCAAGATCAGCGGAAAGAGG

CTGAACATTATGGCTTACGATTACGCAAGATTGGATGGTGAGGGCCACAGGTGGTGGAAG

GGCGGCCGTCTCTCTACTCTGGTGAAGCCTAGGTTCTTCGAAGCGACCGTTGACGATTCT

ACGATTCGTGGACTGTACTTCAAGAATCCTCCTGCATGGTGCTTCGTTTGCAACTGGTGC

CACAACACTGAGATTTCCCGTATGACAGTTGACACCAAAGATGCCGGAGATGGCAGGGCT

GGACGTGCTTACAACACTGATGGTATTGGTTTGGGTTACGTCAAGAACATGACGGTTCTC

AACAGCTACGTGTTCAACCAAGACGACTGTTTCGTTACT

>m_27545

ATGATTGTTGGCTGTTCAGATATTAGCTCTGCGCACAATCAGTGTATATCCCAAAAGAAA

TGCTGGTTATTAACCAACCTTGTTGGTTCCGCATTCGATATTGAGGCCGGCAGGGATGCC

CTTGCAAGGCAACCAAGGCCGTTCGCCCCCTTGAATGTTGGTGTTCCAGACCCAGTCAGA

GCCCTCCGCCACGTACACCCAGTGTTTCGTTCCGTTCCTGAGGACGTTCCCGTGGATGTT

GTTCATGATGAGGTTATTGATGGGGCAACCTTTCGTCGGCTCGCCTTTAGGGCCGTTGTT

TCCGAAGTAGTTGCCGATGACGACGACGCCTATCTCGCTGACGTCCTTGAGCTCGACGTT

GTCGAAGGTGACCCTCTTGACGGAGCCTTTGCCGTTGAGGATGGTCTTGACGCGGACGCC

GATGTTGTTGTTGATAACTTTGCAGTCCTTGATGAGGATGTCTTCGGCTACGTCGCCGTC

GAAGCCGCCGATGACGGCAATCCCGTGGCCTCCGCGGCACTCGTTGTTGGCGAACACGAT

GTTCCTACCCGAGCTGAGGGCCAAACAGTCATCTTGGTTGTTGACTCGGCTGTTGGTGAT

TCTCACATTCTCGGAATGAGCCACATCAAAACCGTCTGTGTTGTGACCACCTTTAGTGTG

TCCAAGTGCGTTATCGATTTCAATATTGTCCATGATGAGATTCTTACAGGCATTGATAGC

CATGACGGTCATAGGTGCGTTCTTGAATACCAAGTCGTTGACGTAGGAGTTCTCAAGCTG

CATGTAGATGAAGGGAGCTGGCTTGATTTTGCCTCCAGTGATACCACCAAGACCGTCCCA

CCAGCGCTGGCCTTCGCCGTCGATAACGTGTCCTGGAAGACCTTTGACGACGTAGTTGGC

GCCTTTGATGATGATCAGGGGGCCCCTCCATTCTTTGTAGCCGAACGAAACTCGTCCAAC

AAACTCAATAGTCGTCCCGTTCCCAAGGCCCTGGAAATCCAATGTCTTGCCGGCAGGGAC

CTGAAGATCTCTCACTCGAACGAGCTTCTGGCCGGTCTTCTTGGCCTCCTCCAGTTGCTG

CATGTTCCACACATCGAAACCAACTGCAGCGTGCAGGACGAAAAACACCCCTCCAAGGAC

TAA

>m_27546

ATGAGGTCAACAATTTTAGTCCTTGGAGGGGTGTTTTTCGTCCTGCACGCTGCAGTTGGT

TTCGATGTGTGGAACATGCAGCAACTGGAGGAGGCCAAGAAGACCGGCCAGAAGCTCGTT

CGAGTGAGAGATCTTCAGGTCCCTGCCGGCAAGACATTGGATTTCCAGGGCCTTGGGAAC

GGGACGACTATTGAGTTTGTTGGACGAGTTTCGTTCGGCTACAAAGAATGGAGGGGCCCC

CTGATCATCATCAAAGGCGCCAACTACGTCGTCAAAGGTCTTCCAGGACACGTTATCGAC

GGCGAAGGCCAGCGCTGGTGGGACGGTCTTGGTGGTATCACTGGAGGCAAAATCAAGCCA

GCTCCCTTCATCTACATGCAGCTTGAGAACTCCTACGTCAACGACTTGGTATTCAAGAAC

GCACCTATGACCGTCATGGCTATCAATGCCTGTAAGAATCTCATCATGGACAATATTGAA

ATCGATAACGCACTTGGACACACTAAAGGTGGTCACAACACAGACGGTTTTGATGTGGCT

CATTCCGAGAATGTGAGAATCACCAACAGCCGAGTCAACAACCAAGATGACTGTTTGGCC

CTCAGCTCGGGTAGGAACATCGTGTTCGCCAACAACGAGTGCCGCGGAGGCCACGGGATT

GCCGTCATCGGCGGCTTCGACGGCGACGTAGCCGAAGACATCCTCATCAAGGACTGCAAA

GTTATCAACAACAACATCGGCGTCCGCGTCAAGACCATCCTCAACGGCAAAGGCTCCGTC

AAGAGGGTCACCTTCGACAACGTCGAGCTCAAGGACGTCAGCGAGATAGGCGTCGTCGTC

ATCGGCAACTACTTCGGAAACAACGGCCCTAAAGGCGAGCCGACGAAAGGTTGCCCCATC

AATAACCTCATCATGAACAACATCCACGGGAACGTCCTCAGGAACGGAACGAAACACTGG

GTGTACGTGGCGGAGGGCTCTGACTGGGTCTGGAACACCAACATTCAAGGGGGCGAACGG

CCTTGGTTGCCTTGCAAGGGCATCCCTGCCGGCCTCAATATCGAATGCGGAACCAACAAG

GTTGGTTAA

>m_31961

ATGAAATTCGTCCTCTTCGCATTGGGTGCGATCGTTGCTGTGGCATCAGCCGTTGATGTT

CACAATCTGGAGCAGCTCGAGGCCGCTAAGAAAGCCAAGGACAAGAACATTGTACTGAAG

AATATCCATGTGCCAGCCGGGAAAACACTGGAACTCCAAGGTCTGGAGCCTGGAACCAAA

GTCACCTTCACTGGACGTGTCACTTTTGGGTACCAAGAATGGAAAGGACATCTCGTGATC

ATCAAAGGACACAAGCTGACTATTGAAGGAAAACCTGGACACTTGATCGATTGTGAGGGA

CACCGTTGGTGGGATGTTCTCGGAGGAAATGGCGGAAAAGTCAAACCCTATGGTATCTAC

GTTCAGCTCACTAATTCCGTTGTCAATGGACTCACCGTGAAGAACACTCCTAAACATGTC

TTCGCCATCAATGGTTGCGAGAACACCGACTTTATTGGAATCACGGTTGACAATGCTGAT

GGACACAAAAAAGGAGGCCACAACACTGATGGA

>m_31964

ATGACCATGCTTTTCTTCACTTCTCCATTATTGAGCCTTCTACTAGTTGTAGGAGTGTCA

CTGGGTTTTGAACTTCAGAGGTTTGAACAACTGGATGATGCTAAACAACAAACATTCATC

AAGGTGAAAAACTTGAACGTTCCAGCAGGAAAGACGTTGGACTTGACTAAATTGAAAGAT

GGGACGACTATTGAATTTGTCGGGCGCACCACTTTTGGTTTCAAAGAGTGGGATGGACCC

TTGGTTAAAATTAGCGGAAAAAATTTGAAAATTGTTGGAGTGAAAGGAAACTTGTTGGAT

GCTGAGGGACAAAGGTGGTGGAATGGAAAAGGTGCCGAACAAGGTTTGAGAAAACCCAGA

ATGTTCGAGGCAATTGTAGACGACTCCATTATCACTGGTCTCAATTTTAAAAACCCACCT

CAAGCTTGCTTTGTATGTAACTGGTGTCACAATGTTCAGATCTCCTGGATAAACATTGAT

GCTAAAGATGGGAGAAATCATCTAGCTTACAATACTGATGGGTTCGGTATCGGATATGCC

AAGAATGTCACATTGACTGACAGTTATGTTTACAATCAAGACGATTGCTTCGTTACAGGA

GCCGGGGAGGATATTCTCGTTGATCGTCTCACTTGCGAGGGAGGAAATGGTATTTCAGTT

GGTTCCCTAGGTGGAGGTGCTAAAGTTGAAAGAGTCACGGTCAGAAACTCCAAAATCATC

GACAACTTGGTCGGTGTCAATGTGAAGACCGGATGGAACGTGAAAGGTTCACTGAAAGAC

ATCACGTTCGACAACATTGAGCTTGTCAACATTCAGCAATTCGGTATCAGCGTTCACGGT

AACGAAGGGCATCCCAACTTCCCTGCTGGTGATCCAACTCCGTTCCCTATTGAAAACTTG

ACCATCAACAACGTGAGAGGAAACGTAAACGGTGCTGGGGCTGCAAACACCTGGGTATGG

GTTGCTCCTGGTAGCGCTAAAAACTGGAAATGGAACTCCAATGTCACTGGTGGGAAGTCA

GCAATGTTCCGCCCACCTCTTCAATGCAAAGGAATTCCAGCTGGTTTGAAAATTCCTTGC

GCTGAGAAATAG

>m_31968

GGAGGCCACAACACTGATGGATTTGATATAGCCAAATCCCATAACATCAAGATCATTAAC

AGCAAAGTAAACAATCAAGACGACTGCTTGGCCATTAACTCAGGTACCAACATTCTCTTC

CAGAACAATATCTGCGAAGGAGGTCATGGTATTGCTGTTGCCGTCGGTGGTTATGATGTC

AACGAAGCCAAGAACATTGTCATCAAGGACTGTCAAGTTATCAAGAACAACATCGGTATC

CGAGTGAAAACTCTGCTCAATGGTAAAGGTATTGTTGATGGAGTCACTTTTGACAACGTT

GTTCTTAAAGACATCAGTGAAATCGGTATTGTCATCATTGGAAACTACCTCAACTCCGGA

CCCAGAGGTGACCCCACCGGCGACCTTCCCATCCGTGGACTGACCATCAACAACGTCCGT

GGAAACGTCCTGAAAAACGGAACCAACATCCATGTCTGGGTGAAGAACGCTGCCAACTGG

AAATGGAACTCCAATGTCGTGGGAGGAACTAGGAAGAAGAATTGC

>m_37990

GAGACCGACAAGAAAGAAGGTCAAATCATGAAGGCTTCAATCATTGCCCTGGGTTGTTTG

GTGGTCGTGGCTTCAGCTATCGACGTCCACAATATTCAACAACTGGATAGTGCCAAGAAA

GGCAACAACAAAGTCATCACCCTGAGGAACATCCAAGTGCCTGCTGGTCAGTCCTTGGAT

CTCGAATCCAACCTAAAGCCTGGCACGACTGTGGAGTTCGCTGGCCGCATCACCTTCGGT

TTCAAAACCTGGAACGGGCCCTTGGTGAGGATCAAGGGGAAGAACCTAAACATCGTCGGA

AAACCAGGACATTCCATCGATGGGGAAGGACACCGCTGGTGGGACGGCAAAGGAGAGCGT

GGTAACACGAAGCCCAACGCTATTTATGTTCAATTGGAGAATTCCAAGGTCACCGGTCTC

TTCCTCAAGAATGCTCCAGCTTGGGGATTTTCCATCAATGGTTGCAAAAACGTCGACTTC

AACCAGATCACTGTTGACAACAAGGACGGAGACCGCAAGGGTGCTTTCAACACGGATGGG

TTTGGCGTAGCTGCATCTAGGAATGTCAAGATAATGAACTCCAAAGTGTACAACCAGGAC

GACTGTTTGGCTCTCCAGTCCGACTGTGACCAAATTTACTTCGACAACAACATTTGTCAA

GGTGGTCACGGTATTGCAGTCATCGGAGGGTACGGAAACCCCAAACCAATCACCAATATC

TTCATCAGAGGATGCCAAGTCATCAAGAACAACATCGGTATCCGTGTGAAGACCATGAGA

GGAGGCAAAGGACTTATCAAGGGCGTCACTTTCGACAATGTCGTTCTGAAGGACATTAGT

GACACCGGAATCATGATCATGGGCAACTACTACAACGGTGGACCACAAGGAGAGCCTACC

AACGGATGCCCCATCACTGGCCTGACCATCAATAACGTGCGTGGTAAT

>m_40846

ATGGTTCCTTCAATTTGCGGGCTTTTCGTGCTGGTCGCTGCTGCTTCAGCGGTTGACGTG

TGGAACCTGCAGCAGCTGGAAGCTGCCAAGAGAGGAAATGATCTCACCATAAACGTCAGG

GACATTTTCGTGCCAGCCGGTCAGACCCTAAACTTCGAGTTTGTGAAGCCTGGAACCACT

ATTGTGTTCAGAGGACGCGTCACTTTCGGCTATAAAGAATGGAGAGGACCTCTCATTATT

CTGAAGGGAAAGAACCTCAAAATCAAAGGAGGAGCCGGGCACATCTTCGACGGTGAAGGT

CGCCGTTGGTGGGACGGAACTGGCACCAACAGTGGTAAGGTGAAGCCGTACATGTTTTAC

GTTCAACTGACAGACTCAAGCGTAAGAGGTTTGACCGTAAAAAACTCTCCTGCTCACACA

TTCGCCATCAACGACTGCCATCACATCTCAATCAACAACGTCATGATTGACAACAGAGAC

GGCAACCGTTTCGGAGGCCACAATACTGATGGGTTTGACATTGCTAAATCCGAGCGCGTT

ATCATCGCCAACAGCACGATTTACAACCAGGATGATTGTTTGGCTATCAACTCCGGTAAT

GACATCACTTTCCAGAGAAACAAGTGTATTGGAGGGCACGGAATAGCCATCGCGGTTGGA

GGATACGATGTTAACCAAGCAACAAACATCAGGATTCGGGGTTGTCGCGCCATACAAACC

AAATACGGAGTTCGCATCAAGACTCTAAGGGGAGGTCGTGGATTGGTTAAAGGAATCAAC

ATCGAAAACATCCTTCTCAAAGACGTCACTGACGCTGGGCTCCTGATTATCGGCAACTAT

CTTAACTCTGGGCCGGGAGGCGAACCAACCGGAGGCATCCCGATCCAGGACTTGCGAGTG

GACAACGTTCGTGGAAATGTCCTAAGCAAAGGAACCAATATACACGTTTTCGTCGCCAAT

GCTTGGAACTGGAGCTGGAATTCCAACATCCAAGGAGGCCAAAGGAGACTGCCTTGCAAG

GGAATTCCTAACGGTCTTCGCATCCCCTGCGGTTAA

>m_42511

ATGACCGAGTCATTAATGAAAACAAATATGAAGCCATTACTCAATGCACTGGGTACCTTC

TTCCTCCTGGTGGCAGTAACAAATGGATTTGAGCTCAAGAGTTTTGACCAACTAGACGCA

GCTAAAAAAAGTTCTGACAAGCATATTGTTATTCGCGATCTCTTCGTGCCTGAAGGCAAA

GTACTGGACTTGTCTAAATTACAGGATGGAACTCTCGTTGAATTCGTGGGACGGGTGACA

TTTGGTTTTAAGGAATGGGATGGCTTCATGGTTTTGATATCAGGAAAGAACATTAGGGTA

GTTGGCAAGCCTGGACACTTAATCGATGGCGAAGGCCATAGATGGTGGGATGGCAAAGGA

GGTAGTGGAGGCAAGAGGAAGCCAAGATTCATGCAGGTTACGTTGGAAAACTCTTTAGTA

TCAGGATTGAACATTAAAAATACTCCAAAAGACGCCTTCGTCGCTAATTTCTGTAAGAAC

GTGCGCATTGAGTATTTGAACGTTGATATCAAGGATGGTGATAGAAAAGGGGGCCACAAT

ACTGATGGAATCGGTGTTGGAGGATCGAGCAACGTCACAGTTTCAAACTGCAAAGTCCAT

AATCAAGATGACTGTTTCTGCATTGGTTCTGGAAGTGATACTGTTTTTGAAAATAATGTT

TGCACTGGTGGGCATGGAATTTCCATTGGGTCTATGGGCGGTGGTAAGAAAGTCGAACGA

CTTCTCGTCAGGAACTGTACTGTCATCAAGAACACCAACGGCATAAGGATCAAGTCGAGA

AAAGGTGAGACCGGTCTGGTCAAAGATGTTACATTTGAAAACATCGAGCTTAGAGAAGTA

ACGCAGTACGGCATCATTATTCACGGAAATTACCCGGACAATGGTCCGAAGAGTGAACCA

ACTCCTTTCCCTATTGAAAATTTGACAATCAACAACGTACGAGGGACTGTCGGCAGAAAA

GCAACCAACATCCTAGTCTGGATCGCTCCAGGAAGCGCAACGAACTGGAAATGGACTTCT

AACATTACTGGTGGAAAGAGGAGACTTTCGTGCAAAGGAGTACCAGCAGGAATCAATATG

CCATGTGGAAAAGTGTGA

>m_43179

ATGGCATCGAAACTCGTGACCTTTACAGGCCTGGTGCTGTGCATTACAGTCGCCGCCGCA

GTGGACATCTGGAGCGTCGATCACCTCGAACAGGCAAAGAAAGGCAATGACCCAGTAATC

AGAGTGCGAGACATCAACGTTCCAGCTGGTCGCACTCTAGATTTCCAAGGTTTAGATGGC

AGAACTATTGAATTCCATGGGCGAGTTACCTTCGGTTACAAGGAATGGCAAGGTCATTTG

ATCATCATCAAGGGTAAGAACATTAAAGTTAAGGGTATGCCTGGCCACTTGATTGATGGT

GAAGGTCACCGCTGGTGGGACAAGTGTGGAGGTAACTGCGGCAAGAAGAAGCCTTTCTTG

ATTTACACTCAGCTTCAGGATTCCACAGTCGATGGGCTTAAGATCAAGAACACTCCTGCC

TGGTGCTTTGCCATCAACGAATGCAACAATGTTCACTACTCCAACATCGATATTGACAAC

AAAGACGGTCATACCAAAGGAGGCCACAACACTGATGGATTCGATGTCCACAAGAGCAGA

AACATCAGGATTTACAATAGCAAGGTCAACAATCAAGACGACTGTTTGGCCATCAACTCT

GGATGGGACATTGTCTTTGAAAACAACGTCTGCGAGGGAGGCCATGGTATCGCTGTTGCT

GTTGGTGGTTATGATGTCAACGAAGCTAAGAACATCTTGATCAGAAACTGCAAAGTTATC

AAAAACAACATTGGAGTCCGCGTCAAAACTTTGTTGAACGGCAAAGGTATTGTTGACGGA

GTTACTTTCGACAACGTTGAATTGAAAGACATTTCTGAGATCGGAATCGTTATAATTGGA

AACTACTTGAATTCTGGCCCACGTGGTGACCCCACTGGAGGAATCCCAATCAAAAACTTG

AACATCAATAACGTGCGTGGTAATGTTCTCCACAACGGAACCAACATTCAGATCAACGTC

GCCCCTGGCAGCCCATCGGGATGGATCTGGAAGTCCAACGTTTGGGGAGGAAAGAAGAAT

CCCAACTGCAAAGGAGTTCCTGGAAACCTCAACAACCCATGCAACTGGTAA

>m_44377

ATGAGAGCGAACGTGGTGATCTTCGGAGTCCTCCTGGTGGTCTTGGCTGCTGCCCACGGG

GCTGTGGTGACGGACTACAACCAATTGGCTGCTGCTAAACAGGGCAACCACATCACGCTG

CGAAACCTGCAGGTCCCAGCTGGAGTTACTTTGGACCTGACGAAGCTCAACCCCGGGACA

ACCGTCGAGTTCGACGGCCGTACGACTTTCGGCTACAAAGAGTGGGCCGGTCCTCTGGTG

AAAGTCAGCGGAAAGAATTTGAGGATCGTCGGTCTCCCTGGGAACCTCCTAGACGGCGAA

GGGAAACGCTGGTGGGATAAACTCGGAGGGAACGGTGGAAAAACGAAACCAAGGTTCATG

GAAGTCAATATTGACGATTCTTCTATTACTGGTTTGAACATCAAAAACCCTCCTGCATGG

TGTTTCGTGGCCAATTACTGTAAAAACGTTCACATCTCAAACGTTAACATCGACATCAAG

GACGGTGATAAGCAAGGAGGCCACAACACTGACGGATTCGGCGTCGGGTACAGCAAAAAT

GTAACAATCCTAAACTGCAAGGTCCACAATCAGGATGATTGCTTCGTCACTGGAGCTGGC

AGTGATATCGTCATCGACAATCTATCCTGCACTGGAGGTCACGGCATTTCAATTGGATCT

TTGGGCCGTGGAGCGGTTGTGGAAAGAGTTTTAGTCAAGAACAGCAAAGTTGCCAGAAAC

ATGGTTGGGATCCGGATAAAATCCACCAGAGGTGAGACTGGAGCTATCAGAGACATCACG

TTTGACAACGTCGAGCTTCAAGGAATCACAAGATACGGTATTATAATCGAAGGGAACTAC

CTGAACTCTGGTTCAGCTGGTGACGCCACTCCGTTCCCAATTGAGAACATCACCATCAAC

AACGTCCGAGGCAGTGTTGTACGCAAGGCCACTAACATCTACGTCAACATCCACCCCACT

AGTGGTAAGAATTGGAAATGGAACTCAAACGTGACCGGAGGCCAGAAAGAACTCAAGTGC

ATTGGTGTTCCTGCTGGTCTCAATATCCCTTGTGGTAAGAAACAGTAA

>m_44495

ACAATAACAGATGCCGTGCAGATAGTTTATAAGCCGGATGGTTTGAAAATATCGTTCTCT

TTTTCTCCATCGTCTTCGCCAGAGGTTGGAGATATCACGGCAAGGATGAACGCTGTCCTA

GGATGTTTGCTGATTGTGGTTGCAACTGCATCTGCGCAATATTTTGAATTGAGAAACGTC

AACCAACTGAATGAAGCGAAAAAATTCCAGAAAATTGTTATTAGAGACCTCCAAGTCCCA

GCTGGTGTCACATTGGACTTGACAAATTTGAAAGATGGAACCACGGTCGAATTCGCCGGA

CGCGTCACATTCGGTTACAAAGAATGGAAAGGACCTCTGGTCAAAGTAGGTGGAAAGAGG

TTAAACATTATAGCTCAACCTCAAGCTAGATTGGACGGCGAGGGCCAAAGGTATTGGAAG

GGTGGTCGTAACACTAAAATGTTGAAACCAAGGTTCTTCGAAGCTATAGTTGACGATTCT

ACGATTCGTGGCCTGTACTTCAAGAACCCTCCTGCACCTTGCTTCCTTTGCAACTGGTGC

CACAACGTCGAAATTTCCCAGATAACAGTTGACGCCAAAGATGCCGGAGACGGCAGAGCT

GGACGTGCTTTCAACACTGATGGTATTAGTTTGGGTTACGTCAGGAACGTTAAAGTTCTC

AACAGCTACGTCTTTAACCAAGACGACTGTTTCGTTACTGGGGGCGGTGAAGACATGCTT

GTCGATAACTTGACCTGCGAAGGAGGTAACGGAATCGGAGTTGGATCACTTGGAAAAGGC

GCTGACGTCAAGCGTTTGACCATCAAAAACAGCAGAGTTATCAACAGTTTGACGGGACTG

AACATCAAGACTGAGGTAAACGCTGTTGGTCTCCACAGGGATGTCACCTTCGACAACATC

GAACTCAAAGATATTCACCAGTATGGAATCACCATTCACGGAAACGAACTTTCCCCTACT

TACCCCCGTGGTGAGCCAACTCTCTTCGCCCTCGAGAACTTGACCATGAGAAACATCAGA

GGAAACATGGTTGGACCTGGAGGTGCCAACGTTTGGATTTGGCTCCATCCCAACAGCGCT

AAGAACTGGAAGTGGCAAAATGTCAACATTAGAGGAGGCAGGAGTGCGATGTGGAAGCCA

CCACTTCAGTGCAAGGGAGTTCCTCCAAATCTTGGAATCCGTTGCGCCGAAAAGTAA

>m_48312

TTAAGCCTGGAACAGTGGTTGGAGTTCTCTCCTAGCATCCATTGGTTTCAAGTGGACCTA

GGTACCTATCTAACTGTGACATTCACATCAATGAAAATGATGTCAAGCGTAGGCACCGTA

GGGGGCCTTTTCTTGGTGATGGCTCTCGCCTCTGCGGTAGATGTTAACAACATGCAACAG

CTGGACGCTGCCAAGAAAGGTAATGACAAACGTATAGTCATCAGGAACCTTCAAGTCCCT

GCTGGAGTCCAGTTGAACCTGGAGAACCTTAAGCCTGGAACAGTGGTTGAGTTTGCTGGC

CGTGTCACTTTCGGATATAAAGAATGGGACGGACCTCTCATCAAAATCTCTGGTAATAAC

ATAAGAGTCGAAGGAAAGCCTGGAAATCTATTGGATGGTGAAGGCGCTCGTTGGTGGGAC

GGCAAAGGAATCTCTGGTGGCAAGAAGAAGCCCAACTTCCTCGAGTTGTACAGGTTGGAC

AATTCGGTCGTAACAGGTTTGAATATAAAAAACGCTCCTCTGAAGATTGTATTGATAAAC

TTCTGCAACCATTTGCAAATCAATAACATTAACTTGGACAATGCTGCTGGTAAAGGCAAA

GCTTTCAACACTGACGGATTTTGTGCTGGCGTTAACAAAGACATTAGAATCAATAACGTT

AGAGTCCATAACCAAGATGATTGTCTCTGCGTACTTGCAACTGACCAGATTTGGTTTGAA

AATAGCGTCTGCACTGGTGGAAACGGAATTTCCATCGGATCCATGGGAGGTGGTTACACA

GTGAAAGGACTTACTGTTAGAAAAGTACAAATCATTGATAGTTTCAATGGTTTGAGGATC

AAGACCAAGAAAAATCAAAACGCCTTGGTGCAAGATGTAACATGGGACGATGTTGTTCTT

AAGGACATTCAACAGAGAGGTATCATCATCCACGGTAACTATCCCAACTGGCGCCCACAG

GACGAACCTGACAACAAAATCCCTATTAGGAACCTTGTCATCAACAACGTTCGCGGAACT

GTGCAGAAAGGTGGCTCCAATATTTGGATCTGGCTTGGCAATGGCGTCGCCTCAAACTGG

AGGGTCAGCAATGTTAAGGTGACAGGCGGTGGTCTTAAACTGGCTTGCAAGGGAATACCA

AAAGGAGTCAACATCGCTTGTGGACAATAA

>m_52832

ATGAAATATTTCTTCATTGCTGTATTTGTGTCTGTGGCCTCAGCAGCTGAAATTTGGAAT

CTCCAGCAGTTAGAGGCCGCCAAGAAAGCTAAAGACAAAAACATCGTTCTAAGAGACATT

CAAGTTCCAGCAGGTCAAACTCTGGAACTTCAAGGTCTGGAAAATGGTACCAGTATCACA

TTTGCTGGACGAATCACATTCGGATACAAAGAGTGGAAGGGACCGTTGGTGATCATCAAA

GGGCATAACTTCCATGTGGAAGGTAAGCCAGGACATGTAATAGACGGTGAGGGACACCGC

TGGTGGGATGGTTTAGGAGGCAATGGCGGCAAAATCAAACCTTACGGAATCTATGTTCAG

CTAACACATTCCAAAGTTAGAAATATCAAAGTAAAGAATTCACCCAAACACTGCTGGGCT

ATCAACGCTTGCCATCACGTAGTGTTTGACGGGATCATTGTCGATGATACTGACGGTCAC

GCTAAAGGAGGGCACAATACTGATGGATTCGACATTGCCAAATCACACCATGTAAAGATA

AAGAACAGCTGGGTCAACAATCAGGATGACTGTTTAGCTTTGAACTCGGGAACTTTCATA

ACGTTTGAGAACAACACCTGTGAAGGAGGGCACGGCATTGCTGTAGCTGTTGGGGGTTAT

GACGAAAACGTCGCCAAACACGTTTATATCAGAAACTGCAAAGTCATTAAGAACAATATC

GGTATCAGAGTGAAGACTCTGTTGAACGGCAAAGGTATTGTAAAGGATATCAATTTCGAG

AATGTGGAGCTTAAGGACATCAGCCAAATCGGGGTCGTCATCATCGGCAACTATCTGAAC

TCAGGGCCGAGGGGGGAACCCACAGGAGACTGTCCCATCCAAGATCTGAAAATTGACAAT

GTTCGAGGGAACGTACTCAGGAATGGAACTAACATTCAGGTCTGGGTAAAGAACGCTTCA

AACTGGAAATGGAAATCTCAAATTGTAGGTGGTACAAAGAAAATACCCTGCCAAGGTATC

CCTAAAGGGGTAAACATACAATGCGGTTAA

>m_53538

GGGAATCTCAGGATTTCTCCGATTCTACGAGACATCAAAGTCGAAATGATGATGTTTAGT

CCTTCAGCTGTCGTAGGCTTTATGGTGGTGACGGTTGCTCTAGCAGTCAATGTGGACAGA

TTTGACCAAATCGAAGCAGCAAAAAAAAGTAACGATAAAGTAATCGTCATCAGAAACCTG

CAAGTTCCTGCTGGGGTGTTGCTGGATTTGCAAAACTTGAAACCTGGTACCACACTTCAG

TTCCAAGGGCGCGTCACGTTTGGTTACAAAGAATGGAAAGGACCTATGGTGAGAATATCG

GGGAAGAACATCATTGTTGAGGGTAAACCTGGACACGTTATAGATGGTGAAGGCGCCCGT

TGGTGGGACGGACTTGGAGGAGCTGGTGGAAAAACCAAACCCACATTCATCGAACTAAAA

TTGGATGATTCAATCGTAAGAGATTTGCACGTTAAAAACACACCAGTACAGATGTTCACA

TCCAACTTCTGCAATAATCTTCTGATCACTAATGTGAACCTTGATAATGAAGACGGCAAG

AACGGTAAGGGCCGCAACACTGATGGATTCGCTGTCGGACTTTCCAAAAACGTCACCGTA

CAAAACAGCCGTGTCTATAATCAAGACGATTGTTTCTGCTGTGGCGCTGGAAGTGACATT

AAGTTCATAAACAACGTCTGTATTGGAGGGAATGGAATCTCCATTGGTTCTATGGGAAAT

AACCGAGTGGTTGAAAGAGTCGAAGCCAGACATTGCCAAATCATCGACAGCTTCAACGGT

ATTCGAATCAAAACGAGAAAGAACGAAAAAGCGCTGGTCAAAGACGTGACATTCGATGAC

ATTGTTCTAAAAGATATCCAACATAGGGGTATTATTGTTCATGGAAATTATCCATCATGG

CGCCCAACTGATGAGCCAACAAACGGTTGTCCAATCCAAAACTTGGTGATCAACAATATT

CGTGGAACAGTCATACCTGGTGGAGCCAACACCTGGATCTGGCTCGCTAAAGGAGTCGCG

TCAGGGTGGAAGGTCAGCAACGTGAATGTTACCGGCGGGAAATTGAAGCTTGAATGCAAA

GGACTTCCTGGCGGCATTGACAAAAACTTCCAGCAAAGATGTGGTCAGATTTAA

>m_59941

ATGAAAAGTAAAACAGCTCCCTGTAGGTCGACGGTTGGGATAAAAATTGTCTCTGCCAGT

ATGACATCCATCGCTGCCTCCTTCGGAGGCCTCCTTCTCGTCCTGGCAGTGTCGTCAGCC

TTCGATCTCAACACCTTCGATCAGCTTGATGCTGCCAAAAAAAGCGCCGACAAGCTCATT

GTCATCAGAAACCTGGTAGTTCCAGCTGGTCAGAAGTTGGACCTGACGAATTTACAACAA

GGAACCGTTATCCGATTCGCCGGCCGCGTGACGTTCGGTTACCAAGAATGGGACGGTACC

ATGATTCAAGTCAAAGGAAAGAACATCAGGGTTGAAGGCAAGCCAGGAAATTTGATCGAT

GGTGAGGGTCACCGTTGGTGGGATAAGAAAGGAGGGAATGGTGGAAAAAAGAAGCCTCGA

TTCATGGAAGTGAATCTAGAAGATTCTATTGTGACTGGTTTGAACATCAAAAATCCTCCA

AGACATTGTTTTGTTGCGAACAACTGCAAAAATGTTCGTATCGAGTATGTCAACATTGAC

ATTAAAGAAGGCGACACAAGGGGAGCCCATAACACTGATGGTTTCGGAGTCGGTGGATCC

CAGAACGTGACGGTAGCTAACTGTAAAGTTCACAACCAGGATGATTGTTTCTGCACTGGA

TCTGGGAGCGATACAGTTTTTGAAAACAATGTCTGCACTGGTGGACATGGAATTTCCATT

GGTTCGATGGGTAACGGACAAAAAGTAGAAAGAGTTCACGTCAGAAACTGTCAAATCATC

AAAAACACCAACGGAATCAGGATTAAATCCAGAAAGGGAGAAACCGGACTCGTACGTGAC

GTCACATTCGAGAACATTGAGCTGAAGGACATCACTCAGTATGGAATCATCATTCAAGGG

AACTACTTGAACGGCGGTCCAAAAGGTGACCCTACTCCTTTCCCAATGGAAAATATTGTC

ATCAAAAACGTGAGGGGAACTGTCAGCAGGAAAGGTACGAATATTTTGGTCTGGGTTGCT

CCTGGAAGTGCCAAAAACTGGCAATGGAATTCGAACATCACCGGAGGGCAGAGAGAAGTC

TCTTGCAAAGGCATACCCCAAGGACTTAACATCCCCTGTGGTAAAAAATGA

>m_66963

ATGATTTCATTTGGGCTTTTGATGTTCTTGGCAGCAGCTTCTGCTGTGGATGTAAACGAT

ATCAAGCAACTGAATGCCGCCAAAAACACTCAGCGCATTACTCTGCGAAACATCAACGTT

CCAGCCGGAGTCACTCTGGATTTGGGCAAACTCAAACCAGGAACCGTAGTTGAATTTGCA

GGACAAATTACATTTGGGTACAAGGAATGGGAAGGGCCTCTTATCTTGATCGGCGGAAAG

AACATCAAGGTTGAAGGCAAACCAGGACATTTGATCAACTGCCAGGGAGAGCGTTGGTGG

GACGGGAAAGGAGGAAATGGAGGGAAGAAAAAGCCAAAGTTCATGGCAGTCAGGCTCACC

GATTCGTCGATTGACGGTCTCCAAGTCAAAAACATACCAGCCCACGGATTTTCGGTTAAC

TCCTGCAAGAACGTGGCCATCTCCAGGATCAACTTGAACGTTGCTGATGGAGACAAGAAA

GGAGGACACAATACTGATGCATTTGATGTAGGTAACTCCGTAGGAATCAAAATCACTGAC

AGCTGGGTCCACAACCAAGATGACTGTTTGGCTATCAATTCTGGAACTGATATTACGTTT

GAGCGCAACACTTGCATTGGAGGACACGGAATTTCTATTGGATCTGTAGGAGGGAGGAAG

AATAACGTCGTTCAGAAGATTAGAGTCCGGCAGTGCAAAGTTATCAATTCCGACAACGGT

ATCCGGATCAAGACTGTAAAAAATACTTCTGGTTCTGTCCAAGATGTATTGTTTGATGAC

GTGGAATTGAAGAATATTGCCAAGCGTGGTATCGTCATCCAAGGCAACTACGAAAACAAG

GGTCCATCAGGCGACCCTACAGGCGGAGTCCCCATCAAAGACCTGACCATCAACAACGTG

CGCGGTAACGTCCTTCCAGCAGGAATTAACGTTTACATTTGGGTTGCCAACGCCTCCAAC

TGGAAATGGAGTAATATAAAAGTCGTAGGTGGAAAGAAAGATCTAGGACAGAAAGGAGTT

CCTAATGGTGTTAAATGGTAA

>m_67886

GAGGATATAATTTCGGAGAGAAACTGCAGGCTGAACAGTGTAATTTATTCCTTTGTGGAG

ACTACAACGACAAAACTGATAATTGGTGACATCATGAATTTGCTTTATTCCATCGGTGGT

TTAATAGTAATTGCCGCTGTTACTGCTGGATTCGATCTCAACAACTTTGCCGACTTGGAC

GCAGCAAAGAAGAGTTCTGACAAGCGTATAGTTATAAAAAACTTGCTAGTTCCGGCTGGT

AAAACACTAGACTTGACCGGATTACAAACTGGTACAGTTATTGAGTTCACTGGGCATGTG

ACGTTCGGTTATGAAGAGTGGGACGGGGAAATGATAAAATTGAAAGGGAAAAACATAACA

GTGATTGGTAAGCCTGGGCATCTTTTGAACGGCGAAGGAAAGCGTTGGTGGGATGGAAAA

GGTGGAAACGGTGGCAAGAGAAAACCTAGATTCATTCAAGTGTCTCTAACTGATTCTACG

ATTACTGGATTGCATATAAAAAATACTCCACGGCATTGTTTCATGATCAACTCAAGTCAA

AATCTGCGAGTGCAAAATACTACAATCGATATCAAAGATGGTGCCAAGCACGGAGGTCAC

AATACTGATGGTTTTGGAGTCAGCAGTTCGCGCAATGTTACCATCTCTAATTCTGTGGTT

TACAATCAAGACGATTGCTTTGCAACAACATCTGGCAGTGACACAGTTTTTGAAAACGCA

AAATGCGTCGGAGGACATGGTATTTCTATTGGTTCTATGGGTTCTGGAAAAGTTGTGGAA

AGAGTGATGATAAGGCATTGCCGGGTATTGGCCAATACCAATGGGATACGCATTAAAACT

AGGAGAGGAGAAACCGGAGCAGTTAAAGACGTTACGTTCCGAGATATAGAAATGAAAGAC

ATATCCAAATACGGAATTGTTATTCAGGGAAACTACTTCAACAGCGGGCCCAGAGGAGAC

CCCACGCCTTTCCCAGTTGAAAATCTGGTGATTGATAATGTTCGAGGCCACGTCATGAAA

TCGGGTGTGAATATTTTGGTCTGGGTAGCTCCGGGAAGTGCTAAAAATTGGACTTGGAGT

TCCAAAATCACTGGGGGACATAAAGAGCAGGAATGCAAAGGTGTACCGATGAATCTAGGC

ATCCGTTGCGGGAAGAAATGA
