# Supplementary material for: Duplication and expression of horizontally transferred polygalacturonase genes is associated with host range expansion of mirid bugs
Source: BMC Evol Biol. 2019 Jan 9;19:12. doi: 10.1186/s12862-019-1351-1 (PMC6327464; doi:10.1186/s12862-019-1351-1)
Supplement: Supplementary file 3 — The coding sequence of PGs identified in this study. (ZIP 71 kb) [file 12862_2019_1351_MOESM3_ESM.zip › Additional file 3-The coding sequence of polygalacturonase genes in Adelphocoris suturalis.docx]

>m_12451

ATGACCATGCTTTTCTTCACTTCTCCATTATTGAGCCTTCTACTAGTTGTAGGAGTGTCA

CTGGGTTTTGAACTTCAGAGGTTTGAACAACTGGATGATGCTAAACAACAAACATTCATC

AAGGTGAAAAACTTGAACGTTCCCGCAGGAAAGACGTTGGACTTGACTAAATTGAAAGAT

GGAACGACTATTGAATTTGTCGGGCGCACCACTTTTGGTTTCAAAGAGTGGGATGGACCC

TTGGTTAAAATTAGCGGAAAAAATTTGAAAATTGTTGGAGTGAAAGGAAACTTGTTGGAT

GCTGAGGGACAAAGGTGGTGGAATGGAAAAGGTGCCGAACGTGGTTTGAGAAAACCCAGA

ATGTTCGAGGCAATTGTAGACGACTCCATTATCACTGGTCTCAATTTTAAAAACCCACCT

CAAGCTTGCTTTGTATGTAACTGGTGTCACAATGTTCAGATCTCCTGGATAAACATTGAT

GCTAAAGATGGGAGAAATCATTTAGCTTTCAATACTGATGGGTTCGGTATCGGATATGCC

AAGAATGTCACATTGACTGACAGTTATGTTTACAATCAAGACGATTGCTTCGTTACAGGA

GCCGGGGAGGATATTCTCGTTGATCGTCTCACTTGCGAGGGAGGAAACGGTATTTCAGTT

GGTTCCCTAGGTGGAGGTGCTAAGGTTGAAAGAGTTACGGTCAGAAACTCCAAAATCATC

GACAACTTGGTCGGTGTCAATGTGAAGACCGGATGGAACGTGAAAGGTTCACTGAAAGAC

ATCACGTTCGACAACATTGAGCTTGTCAACATTCAGCAATTCGGTATCAGCGTTCACGGT

AACGAAGGGCATCCCAACTTCCCTGCTGGTGATCCAACTCCGTTCCCCATTGAAAACTTG

ACCATCAACAACGTGAGAGGAAACGTAAACGGTGCTGGGGCTGCAAACACCTGGGTATGG

GTTGCTCCTGGTAGCGCTAAAAACTGGAAATGGAACTCCAATGTCACTGGTGGGAAGTCA

GCAATGTTCCGTCCACCTCTTCAATGCAAAGGAATTCCAGCTGGTTTGAAAATTCCTTGC

GCTGAGAAATAG

>m_12454

TTAACCGCATTGTATTTTGAATTGAGGAGTGATTTTATTTTGGTTGTTACAAATCAGGAT

CCACAGTTGTCGACGAAGAGAAGTTTAACCGCATTGTATTTTGACGTTGTTGGGGACTCC

CTTGCAGTCCTTCTTCCTAGTTCCTCCCACGACATTGGAGTTCCATTTCCAGTTGGCAGC

GTTCTTCACCCAGACGTGGATGTTGGTTCCGTTGTTCAGGACGTTTCCACGGACGTTGTT

GATGGTCAGTCCACGGATGGGGAGGTCGCCGGTGGGGTCACCTCTGGGTCCGGAGTTGAG

GTAGTTTCCGATGATAACAATACCGATTTCACTGATGTCTTTAAGAACAACGTTGTCAAA

AGTGACTCCATCAACAATACCTTTACCATTGAGCAGAGTTTTCACTCGGATACCGATATT

GTTCTTGATAACTTGACAGTCCTTGATGACGATGTTCTTAGCTTCGTTGACATC

>m_12455

GATGTCAACGAAGCTAAGAACATCGTCATCAAGGACTGTCAAGTTATCAAGAACAATATC

GGTATCCGAGTGAAAACTCTGCTCAATGGTAAAGGTATTGTTGATGGAGTCACTTTTGAC

AACGTTGTTCTTAAAGACATCAGTGAAATCGGTATTGTTATCATCGGAAACTACCTCAAC

TCCGGACCCAGAGGTGACCCCACCGGCGACCTCCCCATCCGTGGACTGACCATCAACAAC

GTCCGTGGAAACGTCCTGAACAACGGAACCAACATCCACGTCTGGGTGAAGAACGCTGCC

AACTGGAAATGGAACTCCAATGTCGTGGGAGGAACTAGGAAGAAGGACTGCAAGGGAGTC

CCCAACAACGTCAAAATACAATGCGGTTAA

>m_14049

ATGGGACCGATGAAGCCGTCTCTCGGATGTGCTTTTCTGTTAATCGCTGTATCATACGGC

TTCGATCTCAACAACTTCGATCAGCTGGACGCCGCCAAAAGAAGCCCCGATAAACGTATC

GTCATCAGAGATCTATTCGTACCGGCAGGCAAGACGTTGGATTTGACAGAATTACAGCCA

GGAACCGTCATTGAGTTTTCAGGTCGAGTCACTTTCGGGTATCAGGAATGGGACGGTTTT

ATGGTTAGATTGAAAGGGAAGGACATCAGGGTGGAAGGCAAACCTGGGAACCTGTTGGAC

GGTGAAGGACATCGCTGGTGGGATGGAAAAGGAGGTAATGGTGGGAAAAGAAAGCCTCGA

TTCATGCAAGTGACTCTGGATGATTCTATTGTAACTGGATTGAACATAAAAAATACTCCC

AAAGACTGTTTTATAGTGAATTGGAGCCATAACCTTCGTGTGGAGCGTATCAACATTGAC

ATTAAAGACGGAGACACGAAAGGTGGACACAATACTGATGGTTTTGGGGTTAGCGGTTCA

ACAAACGTAGTAGTAACAGATTGCCAGGTACATAACCAGGATGATTGCTTTGCTACAACT

TCAGGGAGCGACACGATCTTCGAAAACAGCAAGTGTACGGGCGGGCATGGAATTTCCATC

GGGTCTATGGGCGGCGGTAAGAAAGTCGAACGACTTCTCGTCAGGAACTGTACTGTCATC

AAGAACACCAACGGCATAAGGATCAAGTCGAGAAAAGGTGAGACCGGTCTGGTCAAAGAT

GTTACATTTGAAAACATCGAGCTTAGAGAAGTAACGCAGTACGGCATCATTATTCACGGA

AATTACCCGGACAATGGTCCAAAGAGTGAACCAACTCCTTTCCCTATTGAAAATTTGACA

ATCAACAAC

>m_14052

GGATCCTATTATTGCTGCACTTCATTAATGACCGAGTCATTAATGAACAAAAATATGAAG

CCATTCATATACCTGGGTACCTTCTTCCTCCTTGTGGCCGTAACAAATGGATTTGACCTC

AACAGTTTTGACCAACTAGACGCAGCTAAAAAAAGTTCTGACAAGCATATTGTTATTCGC

GATCTCTTCGTGCCTGAAGGCAAAGTACTGGACTTGTCTAAGTTACAGGATGGAACTCTC

GTTGAATTCGTGGGACGGGTGACATTTGGTTTTAAGGAATGGGATGGCTTCATGGTTTTG

ATAACAGGAAAGAACATTAGGGTAGTTGGCAAGCCTGGACACTTAATCGATGGCGAAGGC

CATAGATGGTGGGACGGCAAAGGAGGGAGTGGAGGCAAGAGGAAGCCAAGATTCATGCAG

GTTACTTTGGAAAACTCTTTAGTATCAGGATTGAACATTAAAAATACTCCTAAAGACGCC

TTCGTCGCCAATTACTGTAAGAACGTGCGCATTGAGTATTTGAACGTTGATATCAAGGAT

GGTGATAGAAAAGGGGGCCACAATACTGATGGAATCGGTGTTGGAGGATCGAGCAACGTC

ACAGTTTCCAACTGTAAAGTCCATAATCAAGATGACTGTTTCTGCATTGGTTCTGGAAGT

GATACTGTTTTTGAAAATAATGTTTGCACTGGTGGGCATGGAATTTCCATCGGGTCTATG

GGAGCCGGGAAAGTTGTGGAAAGGCTGACCGTCAGAAACTGCCGGATTTTGTCTAACACT

AACGGCATTCGAATAAAAACTAGAAGCGGAGAAACGGGAGCAGTGCGTGATGTCACATTT

GAGAACATCGAGCTCAAAGGGATATCTCAGTACGGGATCATAATTCAAGGCAATTATTAC

AATAGTGGGCCGAAAGGTGACCCCACTCCATTCCCTATCCAGAACCTAGTAATTAACAAT

GTTTACGGCAATGTGAGCAGGAAAGGAACTAACATCCTGGTTTGGGTGGACCCTGGTAGC

GTAAGCAACTGGAAATGGAACTCGAAAATTTCAGGAGGGCAGAGAGAACGTGGGTGCAAA

GGTCTTCCACGAAACCTTGATTCTATACGCTGCGGAAAGAAATGA

>m_14054

ATGGGACCGATGAAGCCGTCTCTCGGATGTGCTTTTCTGTTAATCGCTGTATCATACGGC

TTCGATCTCAACAACTTCGATCAGCTGGACGCCGCCAAAAGAAGCCCCGATAAACGTATC

GTCATCAGAGATCTATTCGTACCGGCAGGCAAGACGTTGGATTTGACAGAATTACAGCCA

GGAACCGTCATTGAGTTTTCAGGTCGAGTCACTTTCGGGTATCAGGAATGGGACGGTTTT

ATGGTTAGATTGAAAGGGAAGGACATCAGGGTGGAAGGCAAACCTGGGAACCTGTTGGAC

GGTGAAGGACATCGCTGGTGGGATGGAAAAGGAGGAAATGGTGGGAAAAGGAAGCCTCGA

TTCATGCAAGTGACTCTGGATGATTCTATTGTAACTGGATTGAACATAAAAAATACTCCC

AAAGACTGTTTTATAGTGAATTGGAGCCATAACCTTCGTGTGGAGCGTATCAACATTGAC

ATTAAAGACGGAGACACGAAAGGTGGACACAATACTGATGGTTTTGGGGTTAGTGGTTCA

AGAAACGTAGTAGTAACAGATTGCCAGGTACATAACCAGGATGATTGCTTTGCTACAACT

TCAGGGAGCGACACGATCTTCGAAAACAGCAAGTGTACGGGCGGGCATGGAATTTCCATC

GGGTCTATGGGAGCCGGGAAAGTTGTGGAAAGGCTGACCGTCAGAAACTGCCGGATTTTG

TCTAACACTAACGGCATTCGAATAAAAACTAGAAGCGGAGAAACGGGAGCAGTGCGTGAT

GTCACATTTGAGAACATCGAGCTCAAAGGGATATCTCAGTACGGGATCATAATTCAAGGC

AATTATTACAATAGTGGGCCGAAA

>m_14056

GGATCCTATTATTGCTGCACTTCATTAATGACCGAGTCATTAATGAACAAAAATATGAAG

CCATTACTCAATGCACTGGGTACCTTCTTCCTCCTTGTGGCCGTAACAAATGGATTTGAC

CTCAACAGTTTTGACCAACTAGACGCAGCTAAAAAAAGTTCTGACAAGCATATTGTTATT

CGCGATCTCTTCGTGCCTGAAGGCAAAGTACTGGACTTGTCTAAGTTACAGGATGGAACT

CTCGTTGAATTCGTGGGACGGGTGACATTTGGTTTTAAGGAATGGGATGGCTTCATGGTT

TTGATAACAGGAAAGAACATTAGGGTAGTTGGCAAGCCTGGACACTTAATCGATGGCGAA

GGCCATAGATGGTGGGACGGCAAAGGAGGGAGTGGAGGCAAGAGGAAGCCAAGATTCATG

CAGGTTACTTTGGAAAACTCTTTAGTATCAGGATTGAACATTAAAAATACTCCTAAAGAC

GCCTTCGTCGCCAATTACTGTAAGAACGTGCGCATTGAGTATTTGAACGTTGATATCAAG

GATGGTGATAGAAAAGGGGGCCACAATACTGATGGAATCGGTGTTGGAGGATCGAGCAAC

GTCACAGTTTCCAACTGTAAAGTCCATAATCAAGATGACTGTTTCTGCATTGGTTCTGGA

AGTGATACTGTTTTTGAAAATAATGTTTGCACTGGTGGGCATGGAATTTCCATCGGGTCT

ATGGGCGGCGGTAAGAAAGTCGAACGACTTCTCGTCAGGAACTGTACTGTCATCAAGAAC

ACCAACGGCATAAGGATCAAGTCGAGAAAAGGTGAGACCGGTCTGGTCAAAGATGTTACA

TTTGAAAACATCGAGCTTAGAGAAGTAACGCAGTACGGCATCATTATTCACGGAAATTAC

CCGGACAATGGTCCAAAGAGTGAACCAACTCCTTTCCCTATTGAAAATTTGACTATCAAC

AACGTGCGAGGGACTGTCGGCAGAAAAGCAACCAACATTCTTGTCTGGATCGCTCCAGGA

AGCGCCACGAACTGGAAATGGACTTCTAACATTACTGGTGGAAAGAGGAGACTTTCGTGC

AAAGGAGTACCAGCAGGAATCAATATGCCATGTGGAAAAGTGTGA

>m_15749

CACGCTTCCTCAGGGTCAATCAAGACTGGAATGCTTGTGAACATGGCTCTCACATATATA

GCAGGAAGTTTACTGCTCTCCATCGTAGCAGTCACTGCTGTGGACGTGTGGAACATCCAA

CAACTGGAAGCTGCCAAGAAAGGAAGCGACAGTAAAATATTTATCAGAAATTTGGAGGTT

CCTGCCGGTACCTCACTCAACCTGGAAAATCTAAAACCTGGGACGTACTTGAAATTCGTG

GGCGTCGTTACTTTTGGTTACAAGGAATGGGATGGGCCCCTTATAAAGATATCTGGAAAA

AACATTAAAGTAGAAGGGACTAGGGAAAGCTTAATTGATGGCAATGGAGCTCGCTGGTGG

GATGGCAAGGGGGGAAATGGAGGCAAAAAGAAGCCTAAACTACTATCATTGTGCTTAACA

GACTCAGAGGTCTCTAATTTGAATATTAAAAACTCTCCAGCTCATGGTATTTCTGTAAAT

TGTAAGAACGTCAATATCTACAATATAAACTTTGACAACAAAGATGGCCACTCTAAAGGT

GGGCACAATACTGATGCGTTTGACGTCGGTAATTCAGATCGTGTCACCATAGCTAATTGT

CATGTGGAAAACCAAGATGATTGCTTGGCTGTCAATTCTGGAACCCGGATCGTATTTGAG

AAGAACACCTGCATCGGTGGGCATGGCATTTCCATAGGATCCGTTGGCGGGAGGAGCAAC

AATGTCGTGGATGATGTTATTGTCAGGGATTGCAAGGTCATCAACAACGACAACGGTATC

CGCATCAAGACCAACAAGGATACAACCGGGCTCGTCAAGAACGTCCGTTTCATCAACGTT

GAGCTTCAGAACATCGGAAAAGTCGGGATATCCATTCAAGGGAACTACGCCAACTCCGGG

GCCAAGGGTGACCCAACGGGTGGTGTGCCGATACAGGATTTGCTGATAGACAACGTCTAT

GGGACTGTGAGTCCCCAAGGCGTCAACACAGTTGTCTGGGTAGCAAATGCATCAAGATGG

ACTTGGAGATCCAACGTCAAAGGCGGCAAGAAAAAGGCCGATTGCAAAGGGATCCCAGCA

GGTTTGAAAATTCCTTGCGGCGTTTAG

>m_26424

ATGAAGTATTTCTTCATTGCTGTGTTTGTGTCTGTGGCCTCAGCAGCTGAAATTTGGAAT

CTCCAGCAGTTAGAGGCCGCCAAGAAAGCTAAAGACAAAAACATCGTCCTAAGAGACATT

CAAGTTCCAGCAGGTCAAACTCTGGAACTTCAGGGTCTGGAAAATGGTACCAGTATCACA

TTTGCTGGACGAATCACATTCGGATACAAAGAGTGGAAGGGACCGTTGGTGATCATCAAA

GGGCATAACTTCCATGTGGAAGGTAAGCCGGGACATGTAATAGACGGTGAGGGACACCGC

TGGTGGGATGGTTTAGGAGGAAATGGCGGCAAAATCAAACCTTACGGAATCTATGTTCAG

CTTACACATTCCAAGGTTAGAAATATCAAAGTAAAGAATTCACCCAAACACTGCTGGGCT

ATCAACGGTTGCCGTCACGTAGTGTTTGACGGGATCATTGTAGATGATACTGACGGTCAC

GCTAAAGGAGGGCACAATACTGATGGATTCGACATTGCCAAATCTCACCATGTGAAGATA

AAGAACAGCTGGGTCAACAATCAGGATGACTGTTTAGCTTTGAACTCGGGAACTTTCATA

ACGTTTGAGAACAACACCTGTGAAGGAGGGCACGGCATTGCTGTAGCTGTTGGGGGTTAT

GACGAAAACGTCGCCAAACACGTTTATATCAGAAACTGCAAAGTCATTAAGAACAATATC

GGTATCAGAGTGAAGACTCTGTTGAACGGCAAAGGTATTGTAAAGGATATCAATTTCGAG

AATGTGGAGCTCAAGGACATCAGCCAAACCGGGATCGTCATCATCGGCAACTATCTGAAC

TCAGGGCCGAGGGGGGAACCCACAGGAGACTGTCCCATCCAAGATCTGAAAATTGACAAT

GTTCGAGGGAACGTACTCAGGAACGGAACTAACATTCAGGTCTGGGTAAAGAACGCTTCA

AACTGGAAATGGAAATCTCAAATTGTGGGTGGTACAAAGAAAATACCCTGCCAAGGTGTC

CCTAAAGGGGTAAACATACAATGCGGTTAA

>m_30578

ATGAAAATGATGTCAAGCGTAGGCACCGTAGGGGGCCTTTTCTTGGTGATGGCTCTCGCC

TCTGCGGTAGATGTTAACAACATGCAACAGCTGGACGCTGCCAAGAAAGGTAATGACAAA

CGTATAGTCATCAGGAACCTTCAAGTCCCTGCTGGAGTCCAGTTGAACCTGGAGAACCTT

AAGCCTGGGACAGTGGTTGAGTTTGCTGGCCGTGTCACTTTTGGTTACAAAGAATGGGAC

GGACCTCTCATCAAAATCTCTGGTAATAACATCAGAGTCGAAGGAAAGCCTGGAAATCTA

TTGGATGGTGAAGGCGCTCGTTGGTGGGACGGCAAAGGAATCTCTGGTGGCAAGAAGAAG

CCCAACTTCCTCGAGTTGTACAGGTTGGACAATTCGGTCGTAACAGGTTTGAATATCAAA

AACGCTCCTCTGAAGATTGTATTGATAAACTTCTGCAACCATTTGCAAATCAATAACATT

AACTTGGACAATGCTGCTGGTAAAGGCAAAGCTTTCAACACTGACGGATTTTGTGCTGGC

GTTAACAAAGACATTAGAATCAATAACGTTAGAGTCCATAACCAAGATGATTGTCTCTGC

GTACTTGCAACTGACCAGATTTGGTTTGAAAATAGCGTCTGCACTGGTGGAAATGGAATT

TCCATCGGATCCATGGGAGGTGGTTACACAGTGAAGGGACTTACTGTTAGAAAAGTACAA

ATCATTGATAGTTTCAATGGTTTGAGGATCAAGACCAAGAAAAATCAAAACGCCTTGGTG

CAAGATGTAACATGGGACGATGTTGTTCTTAAGGACATTCAACAGAGAGGTATCATCATC

CACGGTAACTATCCCAACTGGCGCCCACAAGACGAACCTGACAACAAAATCCCTATTAAG

AACCTTGTCATCAACAACGTTCGCGGAACTGTACAGAAAGGTGGCTCCAATATTTGGATC

TGGCTTGGCAATGGCGTCGCCTCAAACTGGAGGGTCAGCAATGTTAAGGTGACAGGTGGT

GGGCTTAAACTGGCTTGCAAGGGAATACCAAAAGGAGTCAACATCGCTTGTGGACAATAA

>m_30580

ATGAAAATGATGTCAAGCGTAGGCACCGTAGGGGGCCTTTTCTTGGTGATGGCTCTCGCC

TCTGCGGTAGATGTTAACAACATGCAACAGCTGGACGCTGCCAAGAAAGGTAATGACAAA

CGTATAGTCATCAGGAACCTTCAAGTCCCTGCTGGAGTCCAGTTGAACCTGGAGAACCTT

AAGCCTGGAACAGTTGTTGAGTTTGCTGGCCGTGTCACTTTTGGTTACAAAGAATGGGAC

GGACCTCTCATCAAAATCTCTGGTAATAACATCAGAGTCGAAGGAAAGCCTGGAAATCTA

TTGGATGGTGAAGGCGCTCGTTGGTGGGACGGCAAAGGAATCTCTGGTGGCAAGAAGAAG

CCCAACTTCCTCGAGTTGTACAGGTTGGACAATTCGGTCGTAACAGGTTTGAATATCAAA

AACGCTCCTCTGAAGATTGTATTGATAAACTTCTGCAACCATTTGCAAATCAATAACATT

AACTTGGACAATGCTGCTGGTAAAGGCAAAGCTTTCAACACTGACGGATTTTGTGCTGGC

GTTAACAAAGACATTAGAATCAATAACGTTAGAGTCCATAACCAAGATGATTGTCTCTGC

GTACTTGCAACTGACCAGATTTGGTTTGAAAATAGCGTCTGCACTGGTGGAAATGGAATT

TCCATCGGATCCATGGGAGGTGGTTACACAGTGAAGGGACTTACTGTTAGAAAAGTACAA

ATCATTGATAGTTTCAATGGTTTGAGGATCAAGACCAAGAAAAATCAAAACGCCTTGGTG

CAAGATGTAACATGGGACGATGTTGTTCTTAAGGACATTCAACAGAGAGGTATCATCATC

CACGGTAACTATCCCAACTGGCGCCCACAAGACGAACCTGACAACAAAATCCCTATTAAG

AACCTTGTCATCAACAACGTTCGCGGAACTGTACAGAAAGGTGGCTCCAATATTTGGATC

TGGCTTGGCAATGGCGTCGCCTCAAACTGGAGGGTCAGCAATGTTAAGGTGACAGGTGGT

GGGCTTAAACTGGCTTGCAAGGGAATACCAAAAGGAGTCAACATCGCTTGTGGACAATAA

>m_33483

ATGCAGCCGTGGTTCCTCTTAATTACGCTCCCGTTGATGAGCGGAATAATTTCAGCTTCA

GAGTATACAATTAGAAGCATCAGCGATGTAAAATACGCCAATAAATACAGCGATATAAAA

ATTTCGAATCTCCATGTGCCAGCCGGAGTTCCACTGGTGTTATTGGGGCTGAAGAATGCA

CGTGTCACATTTGAAGGGACCACCACGTTCGGTTATAAAGAGTGGAAAGGGCATTTGATG

ATGTTCAAAGGGGACAACGTCACCATAACTGGGTCTCCTGGACACTTGATAAACTGCGAG

GGAGAACGATGGTGGGACGGACTGGGTGGTCTCGGCGGAACAAAGAAACCCAAGTTCTTT

GAAGTCCGCCTAAACAATTCGCAAATCTTCGGACTCCAAATCAAAAACACTCCGATGCAC

GCGATTTCCATCAACCACTGCAACAACTTAGTGGTGTCGAACATCATCGTGGACAATCTG

GAAGGCGACTGGAAAGGAGGTCACAATACAGATGGATTCAACGTGTACGAATCAAAAAAC

GTCACCATCAGAAACTGTACTGTGCACAATCAAGATGACTGCATAGCCGTGAAATCTGGA

ATCGACCTGCTTTTCGAGGACCACTTCTGTTCAGGGGGCCATGGGATTTCAATTGGTTCA

GTAGGAGGTCGTAGGGACAATATCGTTGAACGGGCCACTACACGAAATTGCGTCATTCAG

AATTCTGTCAACGGTGTTAGAGTGAAGACAGTCAGGAAGGCCATTGGAAGGGTGTCAAAT

GTGACCTTCGAGAATGTTGCATTGTCGAACATAACCGGATTCGGTATCACAATGCAAGGC

AACTACAACCTGGAACACGGAGATCCGAAAGGCGAACCAACAGGAGGGGTCCCGATCAGT

GGACTCGTCATAAACAACGTGTACGGCACGGTGAACCCAAAAGGAACCAACGTCTGGGTC

TACGTGAAGAACGCCTCAAATTGGAGTTGGGACTTCGACGTAACGGGGGGAACCAAGACT

AGGCGTTGCGAAGGTTTCCCAGATGGAATGACTCCAGTATGTTGA

>m_38348

ATGAGAGCGAACGTGGTGACCTTCGGAGTCGTCCTGGTGGTCTTGGCGGCTGCCCACGCG

GCCGTGGTGACGGACTACAACCAATTGGCTGCTGCTAAACAGGGCAACCACATCACGCTG

CGAAACCTGCAGGTCCCAGCTGGAGTTACTTTGGACCTGACGAAGCTCAACCCCGGGACG

ACCGTCGAGTTTGACGGCCGTACGACTTTCGGCTACAAAGAGTGGGCCGGTCCTCTGGTG

AAAGTCAGCGGAAAGAATTTGAGGATCGTCGGTCTCCCTGGGAACCTCCTAGACGGCGAA

GGGAAACGCTGGTGGGACAAACTCGGAGGAAACGGTGGAAAAACGAAACCAAGGTTCATG

GAAGTCAATATTGACGATTCTTCTATTACTGGTTTGAACATCAAAAACCCTCCTGCATGG

TGTTTCGTGGCCAATTACTGTAAAAACGTTCACATCTCAAACGTTAACATCGACATCAAG

GACGGTGATAAGCAGGGAGGCCACAACACTGACGGGTTCGGCGTCGGGTACAGCAAAAAT

GTAACTATCCAAAACTGCAAGGTCCACAATCAGGATGATTGCTTCGTCACTGGAGCTGGC

AGTGATATCGTCATCGACAATCTATCCTGCACTGGAGGTCACGGCATTTCAATTGGATCT

TTGGGCCGTGGAGCGGTCGTGGAAAGAGTTTTAGTCAAGAACAGCAAAGTTGCCAGAAAC

ATGGTCGGGATCCGGATCAAATCCACCAGAGGTGAGACTGGAGCTATCAGAGACATCACG

TTTGACAACGTCGAGCTTCAAGGAATCACAAGATACGGTATTATAATCGAAGGGAACTAC

CTGAACTCTGGTTCAGCTGGTGACGCCACTCCGTTCCCAATTGAGAACATCACCATCAAC

AACGTCCGAGGCAGTGTTGTACGCAAGGCCACGAACATCTACGTCAACATCCACCCCACT

AGCGGTAAGAATTGGAAATGGAACTCAAACGTGACCGGAGGCCAGAAAGAACTCAAGTGC

ATTGGTGTTCCTGCTGGTCTCAATATCCCTTGTGGTAAGAAATAG

>m_39783

CTCCACAGGGATGTCACCTTCGAAAACATCGAACTCAAAGACATTCACCAGTATGGAATC

AACATTCACGGAAACGAAGGTCCTACTTACCCCTTTGGTGAGTCATCTTTCTTCATCCTC

GAGAACTTGACCGTAAGAAACATCAGAGGAAATATGGTTGGTCCTGGAGGCGCCAACGTC

TGGATTTGGCTTCATCCCGCCAGCGCTAGAAACTGGAGGTGGCAAAATGTCAACATTGTC

GGAGGCAAGAGTGCAATGTGGAGGCCACCACTTCAGTGCAAGGGAGTTCCTCCAAAT

>m_39784

AGTGGAAAGAAGTTAAAAATTATAGGTCATCCTCATGCTAGATTGGACGGCGAGGGCCAA

AGGTATTGGAAGGGTGGTCGTAACACTAAAATGTTGAAACCTAGGTTCTTCGAAGCTATA

GTTGACGATTCTACGATTCGTGGCCTGTACTTCAAGAACCCTCCTGCACCTTGCTTCCTT

TGCAACTGGTGCCACAACGTCGAAATTTCCCAAATAACAGTTGACGCCAAAGATGCCGGG

GACGGCAGAGCTGGACGTGCTTTCAACACTGATGGTATTAGTTTGGGTTACGTCAGGAAC

GTCAAAGTTCTCAACAGCTACGTCTTTAACCAAGACGACTGTTTCGTTACTGGAGGCGGT

GAAGACATCCTTGTGGATAACTTGACCTGCGAAGGAGGTAACGGAATCGGAGTTGGATCT

CTTGGAAAAGGCGCTGACGTCAAGCGTTTGACCATCAGAAACAGCAGAGTTATCAACAGC

TTGACGGGACTGAACATCAAGACTGAGGTGAACGCTGTTGGTCTCCACAGGGATGTCACC

TTCGACAACATCGAACTAAAAGACATTCACCAGTACGGAATCACCATTCACGGAAACGAG

GGTCCAACTTTCCCCACTGGTGAGCCATCTCTCTTCTACCTTGAGAACTTGACCATGAGA

AACATCAGAGGAAACATGGTTGGAACTGGAGGTGCCAACGTCTGGATTTGGCTTCATCCC

GCCAGCGCTAGAAACTGGAGGTGGCAAAATGTCAACGTTGTCGGAGGCAAGAGTGCGATG

TGGAGGCCACCACTTCAGTGCAAGGGAGTTCCTCCAAATCTCGGAATCCGTTGCGCCGAG

AAGTAA

>m_39785

GGTTACAAAGAATGGCGCGGACCTTTGGTCAAGATAGGTGGAAAGAGATTGAACATTATC

GCTCGAAATCGTGCAAGATTGGACGGTGAGGGCCACAGGTGGTGGAATGGTGATCGTCTC

ACTAAAATGTTGAAACCTAGGTTCTTCGAAGCTATAGTTGACGATTCTACGATTCGTGGC

CTGTACTTCAAGAACCCTCCTGCACCTTGCTTCCTTTGCAACTGGTGCCACAACGTCGAA

ATTTCCCAAATAACAGTTGACGCCAAAGATGCCGGGGACGGCAGAGCTGGACGTGCTTTC

AACACTGATGGTATTAGTTTGGGTTACGTCAGGAACGTCAAAGTTCTCAACAGCTACGTC

TTTAACCAAGACGACTGTTTCGTTACTGGGGGCGGTGAAGACATGCTTGTCGATAACTTG

ACCTGCGAAGGAGGTAACGGAATCGGAGTTGGATCTCTTGGAAAAGGCGCTGACGTCAAG

CGTTTGACCATCAGAAATAGCAAAGTTATCAACAGTTTGACGGGACTGAACATCAAGACT

GAGGTGAACGCTGTTGGTCTCCACAGGGATGTCACCTTCGACAACATCGAACTCAAAGAT

ATTCACCAGTATGGAATCACCATTCACGGAAACGAACTTTCCCCTACTTACCCCCGTGGT

GAGCCAACTCTCTTCGCCCTCGAGAACTTGACCATGAGAAACATCAGAGGAAACATGGTT

GGACCTGGAGGTGCCAACGTTTGGATTTGGCTCCATCCCAACAGCGCTAGGAACTGGAAG

TGGCAAAATGTCAACATTAGAGGAGGCAAGAGTTCGATGTGGAAGCCACCACTTGAGTGC

AAGGGAGTTCCTCCACTTGGAATCCGTTGTGCTGAGAAGTAA

>m_40418

ATGGTTCCTTCAATTTGCGGGCTTTTCGTGCTGGTCGCTGCTGCTTCAGCGGTTGACGTG

TGGAACCTGCAGCAGCTGGAAGCTGCCAAGAAAGGAAATGATCTCACCATAAACGTCAGG

GACATTTTCGTGCCAGCCGGCCAGACCCTAAACTTCGAGTTTGTGAAGCCTGGAACCACT

ATTGTGTTCAGAGGACGCGTCACTTTCGGCTATAAAGAATGGAGAGGACCTCTCATTATT

CTGAAGGGAAAGAACCTCAAAATCAAAGGAGGAGATGGGCACATCTTCGACGGTGAAGGT

CGCCGTTGGTGGGACGGAACTGGCACCAACAGTGGTAAGATAAAGCCGTACATGTTTTAC

GTTCAACTGACAGACTCAAGCGTAAGAGGTTTGACCGTAAAAAACTCTCCTGCTCACACA

TTCGCCATCAACGACTGCCATCATATCTCAGTCAACAACATCATGATTGACAACAGAGAC

GGCAACAGGTTCGGAGGCCACAATACTGATGGGTTTGACATTGCTAAATCCGAGCGCGTT

CTCATCGCCAACAGCACGATTTACAACCAGGATGATTGTTTGGCTATCAACTCCGGTAAT

GACATCACTTTCCAGAGGAACAAGTGTATTGGAGGGCACGGAATAGCCATCGCGGTTGGA

GGATACGATGTTAACCAAGCAACAAACATCAGGATTCGAGGTTGTCGCGCCATACAAACC

AAATACGGAGTTCGCATCAAGACTCTAAGGGGAGGTCGTGGATTGGTTAAAGGAATCAAC

ATCGAAAACATCCTTCTCAAAGACGTCACTGACGCTGGACTCCTGATCATCGGCAACTAT

CTTAACTCTGGGCCGGGAGGCGAACCAACCGGAGGCATCCCGATCCAGGACTTGCGAGTG

GACAACGTTCGTGGAAATGTCCTAAGCAAAGGAACCAATATACACGTTTTCGTCGCCAAT

GCCTGGAACTGGAGCTGGAATTCCAACATCCAAGGAGGCCAAAGGAAACTGCCTTGCAAG

GGAATTCCTAACGGTCTTCGCATCCCCTGCGGTTAA

>m_41083

ATGAACGCTGTCCTAGGATGTTTGCTGATTGTGGTTGCAACTGCATCTGCGCAATATTTT

GAATTGAAAAACGTCAACCAACTGAATGAAGCCAAAAAATTCCAGAAAATTGTCATTAGA

AACCTCCAAGTCCCAGCTGGTGTCACATTGGACTTGACCAATTTGAAAGATGGAACCACG

GTCGAATTCGCCGGACGCGTCACATTCGGTTACAAAGAATGGAAAGGACCTATGGTGAAA

ATATCGGGGAAGAACATCATTGTTGAGGGTAAACCTGGACACGTTATAGATGGTGAAGGC

GCCCGTTGGTGGGACGGACTTGGAGGAGCTGGTGGAAAAACCAAACCCACATTCATTGAA

GTGAAATTGGATGATTCAATCGTTAGAGATTTGCACGTTAAAAACACACCAGTACAGATG

TTCAAAGCCAACATCTGCAATAATCTCCTGATCACCAATGTGAACCTTGATAATGAAGAC

GGCAAGAACGGTAAGGGCCGCAACACTGATGGATTCGCAATGGGACTTTCCAAAAACGTC

ACCGTACAAAACAGCCGTGTCTATAATCAAGACGATTGTTTCTGCATTGGCGGTGGAAGT

GACATTAAATTCATAAACAACGTCTGTATTGGAGGGAATGGAATCTCCATTGGTTCTATG

GGAAATAACCGAGTGGTTGAAAGAGTCGAAGCCAGACATTGCCAAATCATCGACAGCTTC

AACGGTATTCGAATCAAAACGAGAAAGAACGAAAAAGCGCTGGTCAAAGACGTGACATTC

GATGACATTGTTCTAAAAAATATCCAACATAGGGGAATTATTGTTCATGGAAATTATCCA

TCATGGCGTCCAACTGATGAGCCAACAAACGGATGTCCAATCCAAAACCTGGTGATCAAC

AATATTCGTGGAACAGTC

>m_41085

TCGCCAGACGTGAGTTCGTGCTTACTGTTTGCTAAGAATCACTTGGTTGGAGAATTCACG

GCAAGGATGAACTCTGTCCTAGGTTGTTTGCTGATTGTGGTTGCAACCGCATCTGCGCAA

TATTTTGAATTGCGAAACGTCAACCAATTGGATGAAGCGAAAAAATTCCAGAAAATTGTC

ATTAGAAACCTCCAAGTCCCAGCTGGTGTCACATTGGACTTGACCAATTTGAAAGATGGA

ACCACGGTCGAATTCGCCGGACGCGTCACATTCGGTTACAAAGAATGGCGCGGACCTCTG

GTCAAGATAGGTGGAAAGAGGTTGAACATTATGGCTTACGATTACGCAAGATTGGATGGT

GAGGGCCACAGGTGGTGGAAGGGCGGCCGTCTCTCTACTCTGGTGAAGCCTAGGTTCTTC

GAAGCTACCGTTGACGATTCTACGATTCGTGGACTGTACTTCAAGAATCCTCCTGCTTGG

TGCTTCGTTTGCAACTGGTGCCACAACACTGAGATTTCCCGTATGACAGTTGACACCAAA

GATGCCGGAGATGGCAGGGCTGGACGTGCTTACAACACTGATGGTATTGGTTTGGGTTAC

GTCAAGAACGTGACGGTTCTCAACAGCTACGTGTTCAACCAAGACGACTGTTTCGTTACT

>m_41087

ATGAACGCTGTCCTAAGTTGTTTGTTGTTTGTGGTTGCAACCGCATCCGCGCAATATTTC

GAATTGAAGAACGTCAATCAGTTGAACGAGGCGAAAAAATACCAGAAAATCGTCATTAGA

GACCTCCAAGTTCCAGCTGGTGTCACGTTGGACTTGTCCAACTTGAGAGAAGGAACCACG

GTTGAGTTTGTCGGACGTGTCACATTCGGTTACAAAGAATGGCGCGGACCTCTGGTCAAG

ATCAGCGGAAAGAGGTTGAACATTATGGCTTACGATTACGCAAGATTGGATGGTGAGGGC

CACAGGTGGTGGAAGGGCGGCCGTCTCTCTACTCTGGTGAAGCCTAGGTTCTTCGAAGCT

ACCGTTGACGATTCTACGATTCGTGGACTGTACTTCAAGAATCCTCCTGCTTGGTGCTTC

GTTTGCAACTGGTGCCACAACACTGAGATTTCCCGTATGACAGTTGACACCAAAGATGCC

GGAGATGGCAGGGCTGGACGTGCTTACAACACTGATGGTATTGGTTTGGGTTACGTCAAG

AACGTGACGGTTCTCAACAGCTACGTGTTCAACCAAGACGACTGTTTCGTTACT

>m_45004

ATTCGAGTCAAAACTTTGCTGAATGGCCACGGTACTGTTGATGGAATCACTTTTGACAAC

GTAGAGCTTACGGATATCTCAGAAACTGGAATAGTTATTATTGGAAATTACTTAAACTCT

GGTCCTAGAGGCTATCCCACTGATGGCATTCCAATTAAAAACCTCAACATCAACAATGTA

CGTGGTAACGTTCTTAATAACGGAACGAACGTGCTGGTTAATGTGTGTCCAGGAACCCCA

TCAGGATGGATCTGGAACTCAAACGTATGGGGTGGAAGGCCAAACGATAAATGTTTGGGC

GTCCCCGATTCAATCGCTAATCCATGCTTGAAGTAA

>m_45314

GGAATAAGTAGGCTGGCGCAGATTGAACCATTAACCATTATGTTCACTTCTTTGGGAGGT

CTCCTTCTTTCCATTACAGTTGCATCTGCAATTGATATCTGGAGTGTGGATCAAATTCCT

CAAGCCAGATACAGTAACGATCCTGTCATCAGGGTGAGAGACATTGTCGTTCCCGCAGGA

ACCGCACTCGATTTCCAAGGATTCGACGGAAAGACCATAGAATTCCACGGACGCGTTACT

TTTGGTTACGAAGAATGGGAAGGGCATTTGATAATCATCAAGGGAAAGAACATGAACATC

AAGGGGATGCCAGGTCACATTATTGACGGCGAAGGTAACCGATGGTGGGATACCTGTGGA

GGGAACTGCGGCAAAAAGAAGCCTTTCATGATCTATACTCAGCTGGAGAATTCATATGTG

GATGGACTCAGGATCAAGAATACTCCTGCATGGTGCTTCGCTATCAATGAATGCACAAAC

GTTCATTACTCAAACATTGAAATCGATAACAAAGACGGACACAGCCAAGGGGGCCACAAC

ACTGATGGATTCGACGTTCACAAAAGTAGGAACATTTGGATCCACGACAGTAAAGTCAAC

AACCAGGACGACTGCTTGGCCATCAATTCCGGGTGGGACATTGTTTTTGAGAACAACGTG

TGTGAAGGAGGTCACGGAATAGCCGTTGCTGTTGGCGGATACGATGTGAACGAAGCTAAA

AATATTTTAATCAAAAACTGCAAAGTAATCAAGAACAACATCGGAATTCGAGTCAAAACT

TTGCTGAACGGCCACGGTACCGTTGATGGAGTCACTTTTGAC

>m_52577

ATGATTTGCCTTGGTCTACTGATGTTCGTGGCCGCAGCTTCTGCTGTAGATGTCAACGAC

ATCAAGCAACTGGATGCTGCCAAAAACTCTCAGCGCATTACTTTGAGAAATATCAACGTT

CCAGCCGGAGTCACTTTGAATTTGGACAAACTCAAACCTGGAACCGTAGTTGAATTCGCT

GGACAAATTACATTCGGGTACAAGGAATGGGAAGGGCCTCTTATCTTGATCGGCGGAAAG

AACATCAAGGTTGAAGGCAAACCAGGACATTTGATCAACTGCCAGGGAGAGCGTTGGTGG

GACGGGCACGGAGGAAATGATGGAAAGAAAAAGCCAAAGTTCATGGCAGTCAGGCTCACC

GATTCGTCGATTGAAGGTCTCCAAGTCAAAAACCTACCCGCCCACGGATTTTCGATTAGC

TCCTGCAAGAACGTGGCCATCTCCAGGATCAACTTGAACGTTGCTGATGGAGACAAGAAA

GGAGGACACAATACTGATGCATTTGATGTAGGTGACTCCGTAGGAATCAGAATCACTGAC

AGCTGGGTCCACAACCAAGATGACTGTTTGGCTATCAATTCTGGAACTGATATTACGTTT

GAGCGCAACACTTGCATTGGAGGACACGGAGTTTCTATTGGATCTGTAGGAGGGAGGAAG

AATAACGTCGTTGACAAGGTTAGAGTCCGTCAGATCAAAGTTATCAATTCCGACAACGGC

ATCCGGATCAAGACTGTGAAAGGAGCTACTGGGTCTGTCAGGGATATCCTGTTTGATGAC

GTGGAATTGAAGAATATTGGTAAGCGTGGTATCGTCATCCAAGGCAACTACGAAAACAAG

GGTCCATCAGGCGACCCTACAGGCGGAGTCCCCATCAAAGACCTGACCATCAACAACGTG

CGCGGTAACGTCCTTCCAGCAGGAACTAACGTTTACATTTGGGTTGCCAACGCCTCCAAC

TGGAAATGGAGTGGAATAAAAATTGTAGGTGGAAAGAAAGACCTTGGACAAAAAGGAGTT

CCCAATGGTGTCAAATGGTAA

>m_52579

AGGATTGCCACAGTCATCTTAGCAGAACACATTTCTGAAAAGAGGCTGGATACCACAATG

ATTTCACTTGGGCTTTTGATGTTGTTGGCAGCAGCTTCTGCTGTGGATGTAAACGATATC

AAGCAACTTAATGCCGCCAAAAACACTCAGCGCATTACTCTGCGAAACATCAACGTTCCA

GCCGGAGTCACTCTGGATTTGAGCAAACTCAAACCTGGGACTGTAGTTGAATTCGCTGGA

CAAATCACGTTTGGGTACAAAGAATGGGAAGGGCCTCTTATCTTGATCGGCGGAAAGAAC

ATCAAGGTTGAAGGCAAACCAGGACATTTGATCAACTGCCAGGGAGAGCGTTGGTGGGAC

GGGAAAGGAGGAAATGGAGGGAAGAAAAAGCCAAAGTTCATGGCTGTCAGGCTCACCGAT

TCGTCGATTAACGGTCTCCAAGTCAAAAACCTACCAGCCCATGGATTTTCGGTTAACTCC

TGCAAGAACGTGGCCATCTCCAGGATCAACTTGAACGTTGCTGATGGAGACAAGAAAGGA

GGACACAATACTGATGCATTTGATGTAGGTGACTCCGTAGGAATCAGAATCACTGACAAC

TATGTCCATAGCCAAGATGACTGTTTGGCTATCAATTCTGGAACTGATATTACGTTCGAG

CGCAACACTTGCATTGGAGGACACGGAATTTCTATTGGATCTGTAGGGGGGAGGAAGAAT

AACGTCGTTGAGAAGGTTAGAGTCCGGCAGTGCAAAGTTATCGATTCCGACAACGGCATC

CGGATCAAGACTGTGAAAGGAGCTACTGGGTCTGTCAGGGATATCCTGTTTGATGACGTG

GAATTGAAGAATATTGGTAAGCGTGGTATCGTCATCCAAGGCAACTACGAAAACAAGGGT

CCATCAGGCGACCCTACAGGCGGAGTCCCCATCAAAGACCTGACCATCAACAACGTGCGC

GGTAACGTCCTTCCAGCAGGAACTAACGTTTACATTTGGGTTGCCAACGCCTCCAACTGG

AAATGGAGTGGAATAAAAATTGTAGGTGGAAAGAAAGACCTTGGACAAAAAGGAGTTCCC

AATGGTGTCAAATGGTAA

>m_53753

ATGAAGGCTTCACTCATTGCCTTGGGTTGCTTGGTGGCTGTTGCTTCAGCTATCGACGTC

AACAATATTCAACAATTGGATGCTGCCAAGAAAGGCAACAACAAAGTCATCACGCTCAGG

AACATCCAAGTGCCTGCTGGCCAATCCTTGGATCTTGAATCCAACCTAAAGCCTGGCACG

ACTGTCGAGTTCGCTGGCCGCATCACCTTCGGTTTCAAAACCTGGAACGGGCCCTTGGTG

AGGATCAAGGGGAAGAACCTAAACATCGTCGGAAAACCAGGACATTCCATCGATGGAGAA

GGACACCGCTGGTGGGACGGCAAGGGACAGCGTGGTAACACGAAGCCCAACGCTATTTAT

GTTCAATTGGAGAATTCCAAGGTCACCGGTCTCTTCCTCAAGAACGCTCCAGCTTGGGGA

TTCTCCGTCAATGGCTGCAAAAACGTCGACTTCAACCAGATCACTGTTGACAACAAGGAC

GGAGACCGCAAGGGTGCTTTCAACACGGATGGGTTCGGCGTAGCTGCATCTAGGAACGTC

AAGATCATGAACTCCAAAGTGTACAACCAGGACGACTGTTTGGCTCTCCAGTCCGACTGT

GACCACATCTACTTCGACAACAACATTTGTCAAGGTGGTCACGGTATTGCAGTTATCGGA

GGGTACGGAAACCCCAAACCAATCACCAATATCTTCATCAGAGGATGCCAAGTCATCAAG

AACAACATCGGAATCCGTGTGAAGACCATGAGAGGAGGCAAAGGACTTATCAAGGGCGTC

ACTTTCGACAATGTCGTTCTGAAGGACATTAGTGACACCGGAATCATGATCATGGGCAAC

TACTACGACGGTGGACCACACGGAGAGCCTACCAACGGATGCCCCATCACTGACCTGACC

ATCAATAACGTGCGTGGTAATGTCCTCAACAACGGAACCAACGTTAGGATTGTTGTCGCT

GGAGATGCCTCTAATTGGAAGTGGAACTCCAACGTCCAAGGAGGAAAGCAAAAGAGAGAG

TGCAAAGGGATGCCCAGAGGAGTTAACGTACCATGCTAA

>m_58361

TACAACGACAAAACTGATAATTGGTGACATCATGAATTTGCTTTATTCCATCGGTGGTTT

AATAGTAATTGCCGCTGTTACTGCTGGATTCGATCTCAACAACTTTGCCGACTTGGACGC

AGCAAAGAAGAGTTCTGACAAACGTATAATTATAAAAAACTTGCTAGTTCCGGCTGGTAA

AACACTAGACTTGACCGGATTACAAACTGGTACAGTTATTGAGTTTACTGGGCATGTGAC

GTTCGGTTATGAAGAGTGGGACGGGGAAATGATAAAATTGAAAGGGAAAAACATAACAGT

GGTTGGTAAGCCTGGGCATCTTTTGAACGGCGAAGGAAAGCGTTGGTGGGATGGAAAAGG

TGGAAACGGTGGCAAGAAAAAACCTAGATTCATGGAAGTGTCTCTAACTGATTCTACGAT

TACTGGATTGCATATAAAAAATACTCCACGGCATTGTTTCATGATCAAGTCAAGTCAAAA

TCTGCGAGTGCAAAATACTACGATCGATATCAAAGATGGTGCCAAGCACGGAGGTCACAA

TACTGATGGTTTTGGAGTCAGCAGTTCGCGCAATGTGACCATCTCTAATTCTGTGGTTTA

CAATCAAGACGATTGCTTTGCAACAACATCTGGCAGTGACACTGTTTTTGAAAATGCAAA

ATGCGTTGGCGGTCATGGGATTTCTATTGGTTCTATGGGTTCTGGAAAAGTTGTGGAAAG

AGTGATGATAAGGCATTGCCGTGTATTGGCCAATACCAATGGGATACGCATTAAAACTAG

GAGAGGAGAAACTGGAGCAGTTAAAAACGTTACGTTTCGAGATATAGAAATGAAAGACAT

ATCCAAATACGGAATTGTTATTCAGGGAAACTACTTCAACAGCGGGCCCAAAGGAGACCC

CACGCCTTTCCCAGTTGAAAATCTGGTGATTGATAATGTTCGAGGCCACGTCATGAAATC

GGGTGTGAATATTTTGGTCTGGGTAGCTCCGGGAAGTGCTAAAAATTGGACTTGGAGTTC

CAAAATCACTGGGGGACATAAAGAGCAGGAATGCAGAGGTGTACCAATGAATCTAGGCAT

CCGGTGCGGGAAGAAATGA

>m_58745

GCCAGTATGACATCCATCGCTGCCTCCTTCGGAGGCCTTCTTCTCGTCCTGGCAGTGTCG

TCAGCCTTCGATCTCAACACCTTCGATCAGCTTGATGCTGCCAAAAAAAGCGCTGACAAG

CTCATTGTTATCAAAAACCTGGTAGTTCCAGCTGGTAAGAAGTTGGACCTGACGAATTTA

CAACAAGGAACCGTCATCCGATTCACCGGCCGCGTGACGTTTGGTTACCAAGAATGGGAC

GGTACCATGATTCAAGTCAAAGGAAAGAACATCAGGGTTGAAGGCAAGCCAGGAAATTTG

ATCGATGGTGAGGGTCACCGTTGGTGGGATAAGAAAGGAGGGAATGGTGGAAAAAAGAAG

CCTCGATTCATGGAAGTGAATCTAGAAGATTCTATTGTGACTGGTTTGAACATCAAAAAT

CCTCCAAGACATTGTTTTGTTGCGAACTACTGCAAAAATGTTCGTATCGAGTATGTCAAT

ATTGACATTAAAGAAGGTGACACAAGGGGAGCCCATAACACGGATGGTTTCGGAGTCGGT

GGATCCCAGAACGTGACGGTAGCTAACTGTAATGTTCACAACCAGGATGATTGTTTCTGC

ACTGGATCTGGGAGCGATACAGTTTTTGAAAACAATGTCTGCACTGGAGGACATGGAATT

TCCATTGGTTCGATGGGTAACGGACAAAAAGTAGAAAGAGTTCACGTCAGAAACTGTAAA

ATCATCAAAAACACCAACGGAATCAGGATTAAATCCAGAAAGGGAGAAACCGGACTCGTA

CGTGACGTCACATTCGAAAACGTTGAGCTGAAGGACATCACTAAGTATGGAATCATCATT

CAAGGGAACTACTTGAACGGCGGTCCAACAGGTGACCCTACTCCTTTCCCAATGGAAAAT

ATTGTCATCAAAAACGTGTGGGGAACTGTCAGCAGGAAAGGTACGAATATTTTGGTCTGG

GTTGCTCCTGGAAGTGCCAAAAACTGGCAATGGAATTCGAAAGTCACTGGGGGGCAGAGA

GAAGTCTCTTGCAAAGGCATACCCCAAGGACTTAACATCCCCTGTGGTAAAAAATGA

>m_62115

ATGAAATTCGTCTTCTTCGCATTGGGTGCGATCGTTGCTGTGGCATCAGCCGTTGATGTT

CACAATCTGGAGCAGCTCGAGGCCGCTAAGAAAGCCAAGGACAAGAACATTGTGCTGAAG

AATATCCAAGTCCCAGCCGGAAGAACACTGGAACTCCAAGGTCTGGAGCCTGGAACCAAG

GTCACATTCACTGGACGTATCACTTTTGGGTACAAAGAATGGAAAGGACCTCTTATGATC

ATCAAAGGACACAAGCTGACTATTGAAGGAAAACCTGGACACTTGATCGATGGAGAGGGA

CACCGTTGGTGGGATGTTCTTGGAGGAAATGGCGGAAAAGTCAAACCCTATGGTATCTAC

GTTCAGCTCACTCATTCCGTTGTCAATGGACTCACCGTGAAGAACTCTCCTAAACATTGT

TTCGCCATCAATGCTTGCGAGAACACCGACTTTATTGGAATCACGGTCGACAATGCTGAT

GGGCACAAAAAAGGAGGCCACAACACTGATGGA

>m_952

GCTCGAACAGGCAAAGAAAGGCAATGACCCAGTAATCAGAGTGCGAAACATCAACGTTCC

AGCTGGTCGCACTCTAGATTTCCAAGGTTTAGATGGCAGAACTATTGAATTCCACGGGCG

AGTTACCTTCGGTTACAAAGAATGGCAAGGTCATTTGATCATCATCAAGGGTAAGAATAT

CAAAGTTAAGGGTATGCCTGGTCACTTGATCGATGGTGAAGGTCACCGCTGGTGGGACAA

GTGTGGAGGTAACTGCGGCAAGAAGAAGCCTTTCTTGATTTACACTCAGCTTCAGGACTC

CACAGTCGATGGGCTTAAGATCAAGAACACTCCTGCCTGGTGCTTTGCCATCAACGAATG

CAACAATGTTCACTACTCCAACATCGATATTGACAACAAAGACGGTCATACCAAAGGAGG

CCACAACACTGATGGATTCGATGTCCACAAGAGCAGAAACATCAGGATTTACAATAGCAA

GGTCAACAATCAAGACGACTGTTTGGCCATCAACTCTGGATGGGACATTGTCTTTGAAAA

CAACGTGTGCGAGGGAGGACATGGTATCGCTGTTGCTGTTGGTGGTTATGATGTCAACGA

AGCTAAGAACATCTTGATCAGAAACTGCAAAGTTATCAAAAACAACATTGGAGTCCGCGT

CAAAACTTTGTTGAACGGCAAAGGTATTGTTGACGGAGTTACTTTCGACAACGTTGAATT

GAAAGACATTTCTGAGATCGGAATCGTTATAATTGGAAACTACTTGAATTCTGGCCCACG

TGGTGACCCCACTGGAGATATCCCAATCAAAAACTTGAACATCAATAACGTGCGTGGTAA

TGTTCTCCACAACGGAACCAACATTCAGATCAACGTCGCCCCTGGCAGCCCATCGGGATG

GATCTGGAAGTCCAACGTTTGGGGAGGAAAGAAGAATCCCAACTGCAAAGGAACTCCTGG

AAACCTCAACAACCCATGCAACTGGTAA

>m_970

ATGAGGTCGACAATTTTAGTCCTAGGAGGGGTGTTTTTCGTCCTGCACGCTGCAGTTGGT

TTCGATGTGTGGAACATGCAGCAACTGGAGGAGGCCAAGAAGACCGGCCAGAAGCTCGTT

CGAGTGAGAAATCTTCAGGTCCCTGCCGGCAAGACATTGGATTTCCAGGGCCTTGGGAAC

GGGACGACTATTGAGTTTGTTGGCCGAGTTTCGTTCGGCTACAAAGAATGGAGGGGCCCT

CTGATCATCATCAAAGGCGCCAACTACGTCGTCAAAGGTCTTCCAGGACACGTTATCGAC

GGCGAAGGCCAGCGCTGGTGGGACGGTCTTGGTGGTATCACTGGAGGCAAAATCAAGCCA

GCTCCCTTCATCTACATGCAGCTTGAGAACTCCTACGTCAACGACTTGGTATTCAAGAAC

GCACCTATGACCGTCATGGCTATCAATGCCTGTAAGAATCTCATCATGGACAACATTGAA

ATCGATAACGCACTTGGACACACTAAAGGTGGTCACAACACAGACGGTTTTGATGTGGCT

CATTCCGAGAATGTGAGAATCACCAACAGCCGAGTCAACAACCAAGATGACTGTCTGGCC

CTCAGCTCGGGTAAGAATATCGTGTTCGCCAACAACGAGTGCCGCGGAGGCCACGGGATT

GCCGTCATCGGCGGCTTCGACGGCGATGTAGCCGAAGACATCCTCATCAAGGACTGCAAA

GTTATCAACAACAACATTGGCGTCCGCGTCAAGACCGTCCTCAACAGCAAAGGCTCCGTC

AAGAGAGTCACCTTCGACAACGTCGAGCTCAAGGACGTCAGTGAGATAGGCGTCGTCGTC

ATCGGTAACTACTTCGGAAACAACGGCCCTAAAGGCGAGCCGACGAAAGGCTGTCCCATC

AATAACCTCATCATGAACAACATCCACGGGAACGTCCTCAGGAACGGAACGAAACACTGG

GTGTACGTGGCGGAGGGATCTGACTGGGTCTGGAACACCAACATTCAAGGGGGCGAACGG

CCTTGGTTGCCTTGCAAGGGCATCCCTGCCGGCCTCAATATCGAATGTGGAACCAACAAG

GTTGGTTAA
