# Supplementary material for: Duplication and expression of horizontally transferred polygalacturonase genes is associated with host range expansion of mirid bugs
Source: BMC Evol Biol. 2019 Jan 9;19:12. doi: 10.1186/s12862-019-1351-1 (PMC6327464; doi:10.1186/s12862-019-1351-1)
Supplement: Supplementary file 3 — The coding sequence of PGs identified in this study. (ZIP 71 kb) [file 12862_2019_1351_MOESM3_ESM.zip › Additional file 3-The coding sequence of polygalacturonase genes in Nesidiocoris tenuis.docx]

>m_23341

CAAATAACAGTTGACGCCAAAGATGCCGGGGACGGCAGAGCTGGACGTGCTTTCAACACT

GATGGTATTAGTTTGGGTTACGTCAGGAACGTCAAAGTTCTCAACAGCTACGTCTTTAAC

CAAGACGACTGTTTCGTTACTGGGGGCGGTGAAGACATGCTTGTCGATAACTTGACCTGC

GAAGGAGGTAACGGAATCGGAGTCGGATCTCTTGGAAATGGCGCTGACGTCAAGCGTTTG

ACCATCAGAAACAGCAGAGTTATCAACAGTTTGACGGGACTGAACATCAAGACTGAGGTG

AACGCTGTTGGTCTCCACAGGGATGTCACCTTCGACAACATCGAACTCAAAGATATTCAC

CAGTATGGAATCACCATTCACGGAAACGAACTTTCCCCTACTTACCCCCGTGGTGAGCCA

ACTCTCTTCGCCCTCGAGAACTTGACCATGAGAAACATCAGAGGAAACATGGTTGGACCT

GGAGGTGCCAACGTTTGGATTTGGCTCCATCCCAACAGCGCTAGGAACTGGAAGTGGCAA

AATGTCAACATTAGAGGAGGCAAGAGTTCGATGTGGAAGCCACCACTTGAGTGCAAGGGA

GTTCCTCCACTTGGAATCCGTTGTGCTGAGAAGTAA

>m_2741

ATGGCATCAAAACTCCTGAGCTTTACAGGCCTGGTGCTGTGCATCACAGTCGCCGCAGCA

GTGGACATCTGGAGCGTCGAGCAGCTCGAACAGGCAAAGAAAGGCAATGACCCAGTAATC

AGAGTGCGAAACATCAACGTTCCAGCTGGTCGCACTCTAGATTTCCAAGGTTTAGATGGC

AGAACTATTGAATTCCACGGGCGAGTTACCTTCGGTTACAAAGAATGGCAAGGTCATTTG

ATCATCATCAAGGGTAAGAATATTAAAGTTAAGGGTATGCCTGGACACTTGATTGATGGT

GAAGGTCACCGCTGGTGGGACAAGTGTGGAGGTAACTGCGGCAAGAAGAAGCCTTTCTTG

ATTTACACTCAGCTTCAGGACTCCACAGTTGATGGGCTTAAGATCAAGAACACTCCTGCC

TGGTGCTTTGCCATCAACGAATGCAACAATGTTCACTACTCCAACATCGATATTGACAAC

>m_28633

TGCTTCGTTACAGGAGCCGGGGAGGATATTCTCGTTGATCGTCTCACTTGCGAGGGAGGA

AACGGTATTTCAGTTGGTTCCCTAGGTGGAGGTGCTAAGGTTGAAAGAGTTACGGTCAGA

AACTCCAAAATCATCGACAACTTGGTCGGTGTCAATGTGAAGACCGGATGGAACGTGAAA

GGTTCACTGAAAGACATCACGTTCGACAACATTGAGCTTGTCAACATTCAGCAATTCGGT

ATCAGCGTTCACGGTAATGAAGGGCATCCCAACTTCCCTGCTGGTGATCCAACTCCGTTC

CCCATTGAAAACTTGACCATCAACAACGTGAGAGGAAACGTAAACGGTGCTGGGGCTGCA

AACACCTGGGTATGGGTTGCTCCTGGTAGCGCTAAAAACTGGAAATGGAACTCCAATGTC

ACTGGTGGGAAGTCA

>m_28906

ATTAGAATCAATAACGTTAGAGTCCATAACCAAGATGATTGTCTCTGCGTACTTGCAACT

GACCAGATTTGGTTTGAAAATAGCGTCTGCACTGGTGGAAATGGAATTTCCATCGGATCC

ATGGGAGGTGGTTACACAGTGAAGGGACTTACTGTTAGAAAAGTACAAATCATTGATAGT

TTCAACGGTTTGAGGATCAAGACCAAGAAAAATCAAAACGCCTTGGTGCAAGATGTAACA

TGGGACGATGTTGTTCTTAAGGACATTCAACAGAGAGGTATCATCATCCACGGTAACTAT

CCCAACTGGCGCCCACAAGACGAACCT

>m_50643

ATGGAACGTTTGGTTTTCGCCCTTGGGCTCTGTCTGGCAATCATCCAGACCATTATTGCC

AATCCAGTCGAAGTCAACAATATCAACCAACTTCAGAATGCGAAGAAATCGAAACACATC

ATCTTGAAGAATGTTGTCGTTCCTGCTGGAGTCAGCTTGGACCTGACTGGCCTTCAACCG

GGTACCGTAGTCGAATTCGATGGAGTTACCAAGTTCGGCTACAAAGAATGGAAAGGACCG

CTCATCAAAACCCAAGGGTCGGGCATCACGATTCGTGGAAGGAACGGACATTTGATTGAC

TGTGAAGGCAGACGATGGTGGGACGGCAAAGGAGGAAACGGTGGTAAAACCAAGCCCCAG

TTCTTCCACATCGTGCTTACTGACTCGAAGATCGAACATCTGAACGTGAAGAACATTCCA

GTCCACGGTTTTGCCATCAACGGAAAGAACATCTACATTGCGTATGTAAGGATTGATAAC

CTCGACGGCCGCACCCAAGGAGGCCACAACACCGACGGCTTTGATGTCGCTAATGCAGAA

AACATTCGGATCACCAATTGCTACGTTGATAATCAGGACGATTGCCTCGCTTTGAACTCC

GGTAAGAACATCCTGTTCGAGTACAACATCTGCAAGGGAGGACACGGAATCTCCATTGCC

CCTTGGGGACACAGTTTCGATGAAGTTCGAGACGTTCGCGTCATCGGTTGCCAGGTGCTC

GACTCTCGTGTTGGCATCCGTGTCAAGACTGGTGCAGACGCTCGTGGTATCGTCAAGAAT

GTTACGTACGACAACATTGTTCTCCAAAACATTCGAGTCAGCGGCATTATTGTCCATGGT

AACTACAGGAACACTGGCCAAATGGGTAAGCCAACTGCTGGAGTCCCTATCGAAGACTTG

ACCATCAATAACGTGCGCGGTAACGTCCTAAAAGACGGAACCAACATCCAAATCTACGTG

GCAGATGGTATGGCAAGGAATTGGAAATGGAGCCAAATCAACGTGAATGGTGGAACCAGG

AAAGTTGAATGCGGTGGAAAACCAAACAACGTCCACGTCCAGTGCGGCTGA

>m_57974

TCTCAAGAGAGGATTGATACCATAATGATTTGCCTTGGTCTACTGATGATCGTGGCCGCA

GCTTCTGCTGTAGATGTCAACGATATCAAGCAACTGGATGCTGCCAAAAACTCTCAGCGC

ATTACTTTGAGAAATATCAACGTTCCAGCCGGAGTCACTTTGAATTTGGACAAACTCAAA

CCTGGAACCGTAGTTGAATTTGCAGGACAAATTACATTCGGGTACAAGGAATGGGAAGGG

CCTCTTATCTTGATCGGCGGAAAGAACATCAAGGTTGAAGGCAAACCAGGACATTTGATC

AACTGCCAGGGAGAGCGTTGGTGGGACGGGAAAGGAGGAAATGGAGGGAAGAAAAAGCCA

AAGTTCATGGCTGTCAGGCTCACCGATTCGTCGATTGACGGTCTCCAAGTCAAAAACATA

CCAGCCCACGGATTTTCAGTTAACTCCTGCAAGAACGTGGCCATCTCCAGGATCAACTTG

AACGTTGCTGATGGAGACAAGAAAGGAGGACACAATACTGATGCATTTGATGTAGGTGAC

TCCGTAGGAATCAGAATCACTGACAGCTGGGTCCACAACCAAGATGACTGTTTGGCTATC

AATTCTGGAACTGATATTACGTTTGAGCGCAACACTTGCATTGGAGGACACGGAGTTTCT

ATTGGATCTGTAGGAGGGAGGAAGAATAACGTCGTTGACAAGGTTAGAGTCCGTCAGATC

AAAGTTATCAATTCCGACAACGGCATCCGGATCAAGACTGTGAAAGGAGCTACTGGGTCT

GTCAGGGATATCCTGTTTGATGACGTGGAATTGAAGAATATTGGTAAGCGTGGTATCGTC

ATCCAAGGCAACTACGAAAACAAGGGTCCATCAGGCGACCCTACAGGCGGAGTCCCCATC

ACAGACCTGACCATCAACAACGTGCGCGGTAACGTCCTTCCAGCAGGATGTAATGTTTAC

GTTTGGGTCGCCAACGCCTCCAACTGGAAATGGAGTGGAGTGAAAATTGCAGGT

>m_85860

GCTGATGGGCACAAAAAAGGAGGCCACAACACTGATGGATTCGATGTCCACAAGAGCAGA

AACATCAGGATTTACAATAGCAAGGTCAACAATCAAGACGACTGTTTGGCCATCAACTCT

GGATGGGACATTGTCTTTGAAAACAACGTGTGCGAGGGAGGACATGGTATCGCTGTTGCT

GTTGGTGGTTATGATGTCAACGAAGCTAAGAACATCGTCATCAAGGACTGTCAAGTTATC

AAGAACAATATCGGTATCCGAGTGAAAACTCTGCTCAATGGTAAAGGTATTGTTGATGGA

GTCACTTTTGACAACGTTGTTCTTAAAGACATCAGTGAAATCGGTATTGTTATCATCGGA

AACTACCTCAACTCCGGACCCAGAGGTGACCCCACCGGCGACCTCCCCATCCGTGGACTG

ACCATCAACAACGTCCGTGGAAACGTTCTGAAC
